# Long-term atmospheric deposition of nitrogen, phosphorus and sulfate in a large oligotrophic lake

**DOI:** 10.7717/peerj.841

**Published:** 2015-03-19

**Authors:** Bonnie K. Ellis, James A. Craft, Jack A. Stanford

**Affiliations:** Flathead Lake Biological Station, University of Montana, Polson, MT, USA

**Keywords:** Atmospheric loading, Atmospheric deposition, Flathead Lake, Aerosols, Limiting nutrient, Aerosol index, Air inversions, Air quality, Phosphorus, Nitrogen, Sulfate

## Abstract

We documented significantly increasing trends in atmospheric loading of ammonium (NH_4_) and nitrate/nitrite (NO_2/3_) and decreasing trends in total phosphorus (P) and sulfate (SO_4_) to Flathead Lake, Montana, from 1985 to 2004. Atmospheric loading of NO_2/3_ and NH_4_ increased by 48 and 198% and total P and SO_4_ decreased by 135 and 39%. The molar ratio of TN:TP also increased significantly. Severe air inversions occurred periodically year-round and increased the potential for substantial nutrient loading from even small local sources. Correlations between our loading data and various measures of air quality in the basin (e.g., particulate matter <10 µm in size, aerosol fine soil mass, aerosol nutrient species, aerosol index, hectares burned) suggest that dust and smoke are important sources. Ammonium was the primary form of N in atmospheric deposition, whereas NO_3_ was the primary N form in tributary inputs. Atmospheric loading of NH_4_ to Flathead Lake averaged 44% of the total load and on some years exceeded tributary loading. Primary productivity in the lake is colimited by both N and P most of the year; and in years of high atmospheric loading of inorganic N, deposition may account for up to 6.9% of carbon converted to biomass.

## Introduction

Atmospheric deposition can be a significant source of nitrogen (N) and phosphorus (P) to lakes (Likens et al., 1985; Bergstrom & Jansson, 2006; Morales-Baquero, Pullido-Villena & Reche, 2006). Increased N and/or P inputs can speed eutrophication (Wetzel, 2001), alter lake phytoplankton community composition (Baron et al., 2000; Wolfe, Baron & Cornett, 2001), and alter biogeochemical cycling, trophic dynamics and biological diversity (Elser et al., 2009).

Globally, atmospheric deposition of N actually exceeds riverine inputs to surface waters (Paerl, 1997) and has become the dominant reactive N distribution process (Galloway et al., 2008). The cause for the increasing N deposition in the western U.S. has been attributed to rapidly increasing population, increasing urbanization and vehicle use and increasing size of concentrated animal feeding operations (Fenn et al., 2003a). However, N deposition data and source information are lacking for most areas in the western U.S. and Fenn et al. (2003b) emphasize the need for long-term catchment studies from western ecosystems that include N inputs from dry deposition.

Flathead Lake is the 79th largest lake in the world (Herdendorf, 1982) and the largest freshwater lake in the western U.S. outside of Alaska. Although the lake is among the cleanest in the world (Stanford & Ellis, 2002), primary productivity has steadily increased since 1977, coherent with a 41% increase in human population on the lake shore and areas immediately upstream (Ellis et al., 2011). Previous studies have shown that primary production in the lake is colimited by N and P (Dodds & Priscu, 1989; Dodds & Priscu, 1990; Spencer & Ellis, 1990; Spencer & Ellis, 1998); therefore, increasing loading rates and/or shifting the N:P ratio may have significant consequences for lake water quality.

The primary objective of this study was to determine whether atmospheric deposition was a significant source of N, P, and SO_4_ to Flathead Lake for the period 1984–2004 and whether significant increasing trends in loading occurred. Sulfate (SO_4_) loading is a strong indicator of acidification, which may be a water quality concern in addition to N and P as plant growth nutrients. The second objective was to identify probable sources of atmospheric loads and to examine whether the propensity for air inversions in the valley were affecting atmospheric contributions. We limited the analysis to 1984–2004 because source data were available for that period and because the collection site and collector design was changed in 2005. This study provides a long-term history of atmospheric deposition (wet plus dry) of N, P, and SO_4_ to a large oligotrophic lake in a rural but growing area of the western U.S. and compares results to other lakes in the region and beyond.

## Material and Methods

### Study area

**Figure 1:**
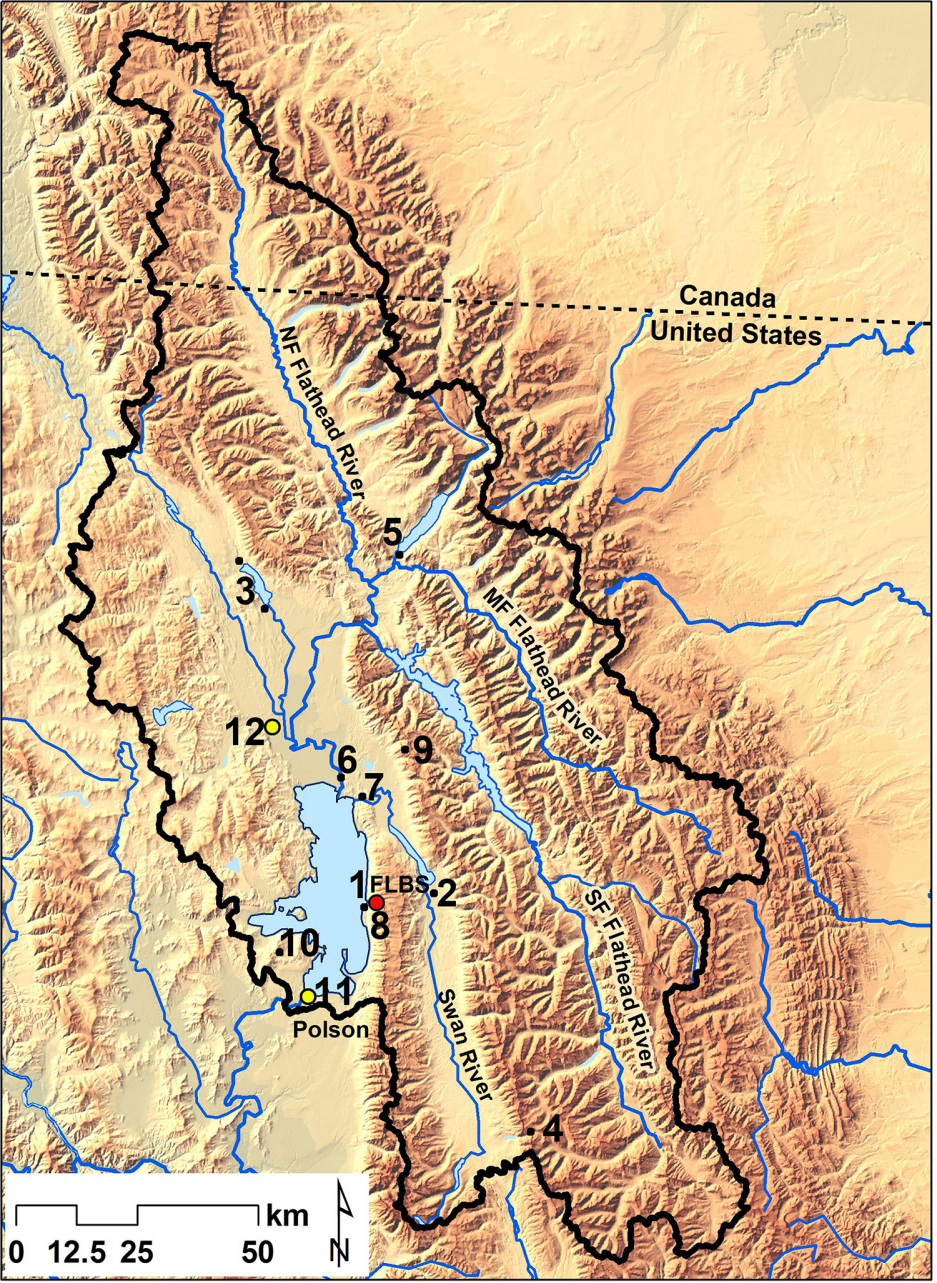
Monitoring sites in the Flathead Lake watershed. Flathead Lake watershed (black outline) in northwest Montana and southeast British Columbia. The Flathead Lake Biological Station (FLBS) on the east shore of Flathead Lake is denoted by the red circle and is the location of the Flathead Lake atmospheric deposition collector and the NOAA weather station (1). Other numbered monitoring sites include FLBS atmospheric deposition collectors on Swan Lake (2), Whitefish Lake (3), and on Pendant Pass (4), National Atmospheric Deposition Program (NADP) collector on McDonald Lake (5); the primary tributary sites on the mainstem Flathead River (6) and Swan River (7), and a small shoreline tributary Yellow Bay Creek (8); Natural Resources Conservation Service Noisy Basin SNOTEL site (9), the Interagency Monitoring of Protected Visual Environments (IMPROVE) site (10), Montana Department of Environmental Quality aerosol coarse and fine particulate monitoring sites in the cities of Polson (11) and Kalispell (12).

Flathead Lake is oligotrophic on par with Lakes Tahoe and Superior (Wetzel, 2001; see Table S1 for comparative metrics). Flathead Lake receives runoff from 18,290 km^2^ in northwestern Montana, USA, and southeastern British Columbia, Canada (see Fig. 1). The primary tributary is the 6th order Flathead River that drains Glacier National Park and other protected or managed areas; relatively pristine alpine areas, upland forests, and intermountain grasslands make up 61% of the catchment. The remainder is mostly in the expansive valley that also contains the lake; this area is extensively modified by silviculture, agriculture, urban, and exurban land uses. About 105,000 people live within the catchment but tourism adds substantially more. Runoff peaks (two orders of magnitude over base flow) with spring snow melt, usually in late May or early June, which entrains suspended sediments, mainly fine clays, in the water column of the lake. Aerosols in the airshed are substantially increased during periods of air stagnation caused by temperature inversions. Entrainment of dust from agricultural fields and rural roads and smoke from forest fires and household and industrial combustion occurs (Stanford & Ellis, 2002). The long-term (1939–2004) average annual precipitation was 56 cm at Flathead Lake (Fig. S1).

### Atmospheric deposition at Flathead Lake

Collection of atmospheric deposition at Yellow Bay on the east shore of Flathead Lake (Fig. 1) began in 1982 and continues to present day. This study describes the results for the period 1982 to 2004 during which the collectors composited wet- and dry-fall. After 2004, wet- and dry-fall were collected and measured separately using an Aerochem Metrics wet-dry device. A modification of the collector described by Lewis & Grant (1978) was used from 1982 to 2004 and consisted of duplicate high-density polyethylene funnels (33 cm diameter, 17 cm depth) with Teflon© spikes around the top that prevented birds from landing and the use of high-density polyethylene collection cylinders (38 cm wide by 69 cm depth) during winter, which reduced snow loss during extreme wind and snow events. The collectors were located on a 10 m tower 30 m from the shoreline at the Flathead Lake Biological Station per the guidelines of the National Atmospheric Deposition Program (NADP) (Bigelow, 1984). All collectors were routinely cleaned with ultrapure deionized water and a soft brush, rinsed with 10% HCl, soaked in Type 1 deionized water. Collectors tested negative for potential leaching of Cl, N, and P. Atmospheric deposition samples were obtained after each major precipitation event, usually 30 times per year over the entire study period.

To examine potential underestimation of dry deposition by our use of dry collectors (as opposed to entrainment in water), we conducted an experiment utilizing identical collectors (in duplicate; 39 cm diameter, 3 cm depth) with and without Type 1 deionized water and compared results over 4–5 days. The wet collectors collected 48X more NH_4_ and 6.6X more TN than the dry collectors during smoky periods but results were similar otherwise (Table S2). Although the experimental collectors were not identical to the atmospheric collector, results suggest that NH_4_ and TN may be underestimated by the atmospheric deposition collector during smoky conditions, which underscores the rationale herein for limiting the analysis of the time series prior to collector change in 2005.

Samples were collected in bottles that were acid-washed and soaked in Type 1 deionized water. Analytical protocols for P and N forms and SO_4_ followed standard methods (Table S3; see also Baumann & Stanford, 2014); 0.45 µm membrane filters were used for dissolved forms. All sample data and quality control information are electronically archived by the FLBS Data Manager in the Biological Station’s digital data storage and retrieval system. To obtain time series estimates of bioavailable-P, we reduced measured concentrations of TP in atmospheric deposition by 24.4% in accordance with experiments that showed Flathead phytoplankton can only assimilate 75.6% of TP (Ellis & Stanford, 1986).

Time series N, P, and SO_4_ concentrations were weighted by precipitation during each sampling interval to calculate loads. For determination of long-term trends, concentrations and loads were natural log transformed and the data analyzed in the “classical decomposition model,” in the statistical software program ITSM 2000 (Brockwell & Davis, 2002); the model removes seasonality using a standard *d*-component moving average function. Residuals from the deseasonalized data were examined with linear regression (SPSS statistics program) to demonstrate significant trends in each data time-series.

### Comparative deposition data

Precipitation-weighted mean concentration of nutrients and sulfate in atmospheric deposition at Flathead Lake were compared to data we collected using identical methods at two other Flathead Basin lakes located upstream from Flathead Lake: Whitefish Lake (elevation 913 amsl), 63 km NW but in the same valley as Flathead Lake, and Swan Lake (934 amsl), also in a valley bottom but 14 km east and segregated from Flathead Lake by the Swan mountain range. We also installed a pair of collectors located at 2,042 amsl at Pendant Pass in the Swan Range about 57 km SE of Flathead to obtain high elevation data. Deposition data also were available from a National Atmospheric Deposition Program (NADP) site located at McDonald Lake in Glacier National Park, 71 km upstream from Flathead Lake. Finally, we compared atmospheric deposition of P at Flathead to that reported for other lakes and aquatic systems around the U.S. and Europe.

### Sources of aerosols

Comparison of atmospheric loads to the amount of aerosols (measured using aerosol coarse and fine particulates), the composition of aerosols (determined from the Interagency Monitoring of Protected Visual Environments (IMPROVE) chemical data), emission data from point and area sources, and occurrence of air inversions and forest fires helped identify probable sources of atmospheric loads. Aerosol coarse and fine particulate data (PM10 and PM2.5) was obtained for sites near Flathead Lake at Polson (south end of the lake) and Kalispell (north), Montana (Fig. 1; data from Montana Department of Environmental Quality for the period 1988–2001). Both sites used high volume samplers early on and then switched to a tapered element oscillating microbalance. Additional aerosol particle and chemical data was obtained from the IMPROVE database for a site installed near Polson (elevation 1,552; Fig. 1) in 2002 (see Malm et al., 1994 and Sisler et al., 1996 for analytical methods and calculation of aerosol types). Source and deposition data were examined in relation to time periods of air stagnation (inversions) based upon National Oceanic and Atmospheric Administration (NOAA) and Natural Resources Conservation Service Snow Telemetry (SNOTEL) air temperature data and in relation to duration and area burned by local (within basin) forest fires (US Forest Service and Montana Department of Natural Resources and Conservation data). Aerosol emission of NOx, SO_2_, NH_3_, and PM10 from point and area (nonpoint, on-road, off-road) sources for the 11 counties surrounding Flathead Lake were obtained from USEPA Emission Inventory and Analysis Group, Triangle Park, North Carolina. We also obtained aerosol index (AI) data from the Laboratory for Atmospheres, Goddard Space Flight Center, Greenbelt, Maryland, for the same time period. The presence of tropospheric aerosols can be detected by comparing the measured ratio of the backscattered radiances at two wavelengths (340 and 380 nm measured with the total ozone mapping spectrometer (TOMS)) to a calculated ratio based on a Rayleigh-scattering model atmosphere bounded by Lambertian surfaces (Herman et al., 1991). The logarithmic difference between measured and calculated ratios is defined as the aerosol index. Thus, correlations of AI with our deposition data indicated that incoming aerosols could be derived from areas far from Flathead Lake, as opposed to within basin sources.

### Loading from tributaries

Grab samples (Van Dorn bottle) were obtained from the two primary tributaries (Flathead and Swan Rivers; Fig. 1) 15 times per year for analyses of N and P concentrations using the same analytical protocols as for atmospheric deposition. Daily discharge data were obtained from the U.S. Geological Survey (USGS) or we monitored flows per USGS procedures (Rantz et al., 1982a; Rantz et al., 1982b). Loading estimates for the small perennial streams around the perimeter of the lake were made using nutrient data from Yellow Bay Creek (Fig. 1; *n* = 24; see Ellis, 2006) and estimated annual discharge obtained by FLBS and Confederated Salish and Kootenai Tribes. Daily precipitation volume to the lake surface was determined from the NOAA site maintained at FLBS.

Daily concentration estimates were interpolated from the time series of measured data and daily loads were calculated by multiplying concentration by flow. Bioavailable P loading also was estimated. Ellis & Stanford (1988) showed that only 8% of the TP entering the lake in the form of fine inorganic sediments during erosive high flow events in the Flathead River is bioavailable for uptake by phytoplankton in the lake. Thus, during base flow, when little or no inorganic sediments were present in the samples, interpolations were directly flow-weighted, but during high flow events when sediments were present in samples, bioavailable-P was predicted as a function of discharge.

## Results and Discussion

### Atmospheric nitrogen deposition and sources

Precipitation-weighted concentrations of N forms were routinely an order of magnitude greater than SRP and TP in the time series data, although with considerable variation (Table 1). Inorganic forms of nitrogen (NH_4_ and NO_2/3_) dominated TN (Table 1). Average annual concentration of NH_4_ was more than twice that of NO_2/3_. When the time series N data were deseasonalized, we observed gradually increasing trends in concentrations of NH_4_ (*p* < 0.0001), NO_2/3_ (*p* < 0.0001), and TN (*p* < 0.05).

**Table 1:** Concentration and range of N, P and **SO_4_** in atmospheric deposition. Precipitation-weighted mean concentration (±1.0 SD) and range of N, P and SO_4_ in atmospheric (wet + dry) deposition collected during the period 1982–2004 (excluding water years 1983, 1984; NH_4_ data unavailable 1982, 1998; SO_4_ data unavailable 1998).

	NH_4_–N (µg L^−1^)	NO_2/3_–N (µg L^−1^)	TN (µg L^−1^)	SRP (µg L^−1^)	TP (µg L^−1^)	SO_4_ (mg L^−1^)
Mean	390 ± 276	187 ± 41	660 ± 344	22.7 ± 22.1	56.8 ± 57.9	0.52 ± 0.10
Range	88–1,204	116–255	336–1,908	1.6–83.1	9.9–245	0.29–0.68

**Figure 2:**
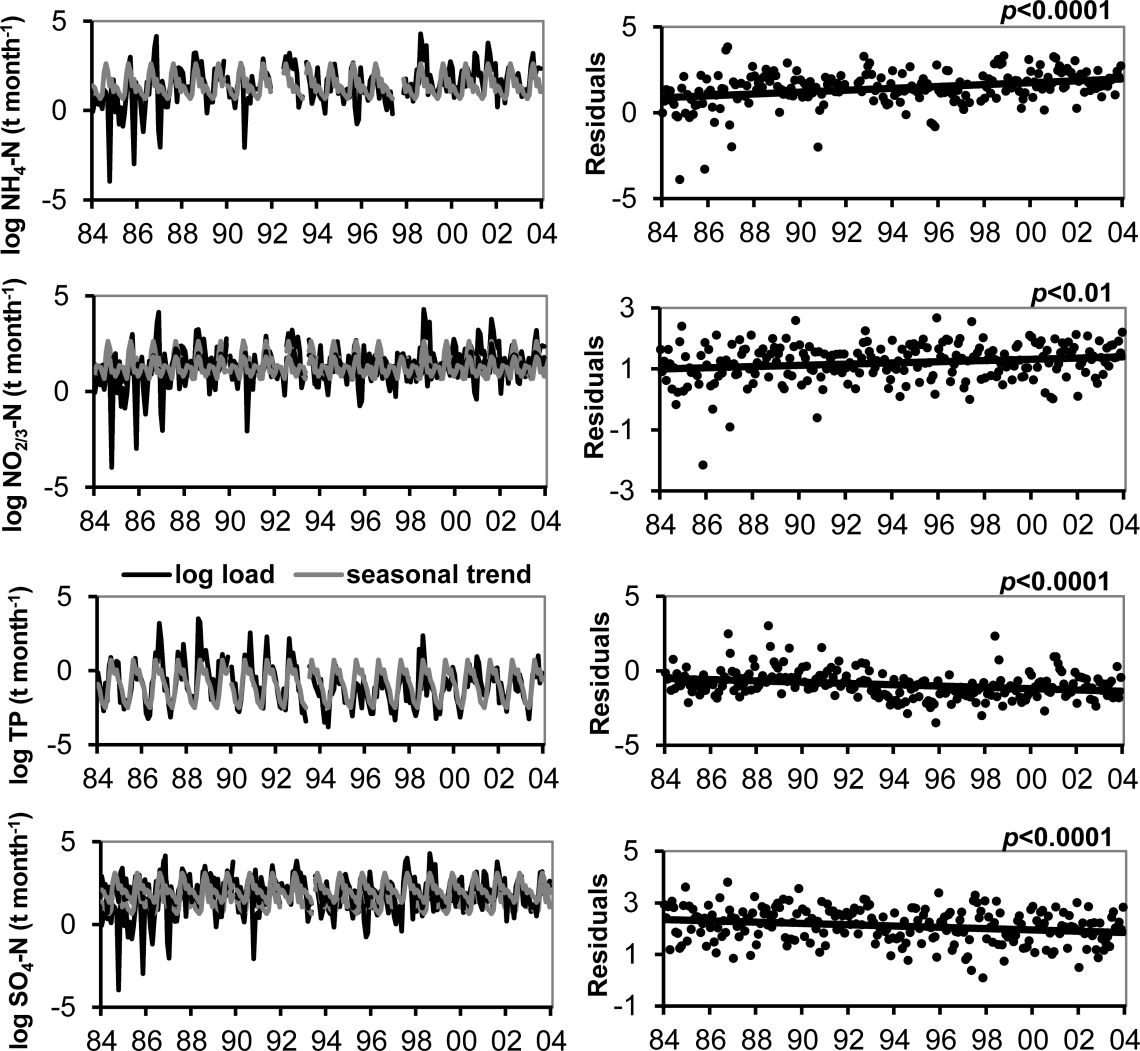
Trends in atmospheric loading of N, P and **SO_4_**. The natural log of atmospheric deposition NH_4_–N,NO_2/3_–N, TP and SO_4_ load (metric tons month^−1^) at the Flathead Lake Biological Station and the best-fit seasonal trend are shown in the graphs at left. A least squares regression of the deseasonalized data (residuals) against date display the linear trend in the graphs at right.

We observed no long-term trend in volume of precipitation, so loading trends in the atmospheric deposition data were substantially driven by concentrations. The increase in NH_4_ concentration in atmospheric deposition resulted in a 198% increase in atmospheric loading of NH_4_ to Flathead Lake from 1985 to 2004 (Fig. 2). Likewise, the increase in NO_2/3_ concentrations produced a 48% increase in atmospheric NO_2/3_ loading (Fig. 2). No significant trend in atmospheric TN loading to the lake was observed. The lack of a trend in TN loading likely was due to a decrease in organic N loading over the period.

**Figure 3:**
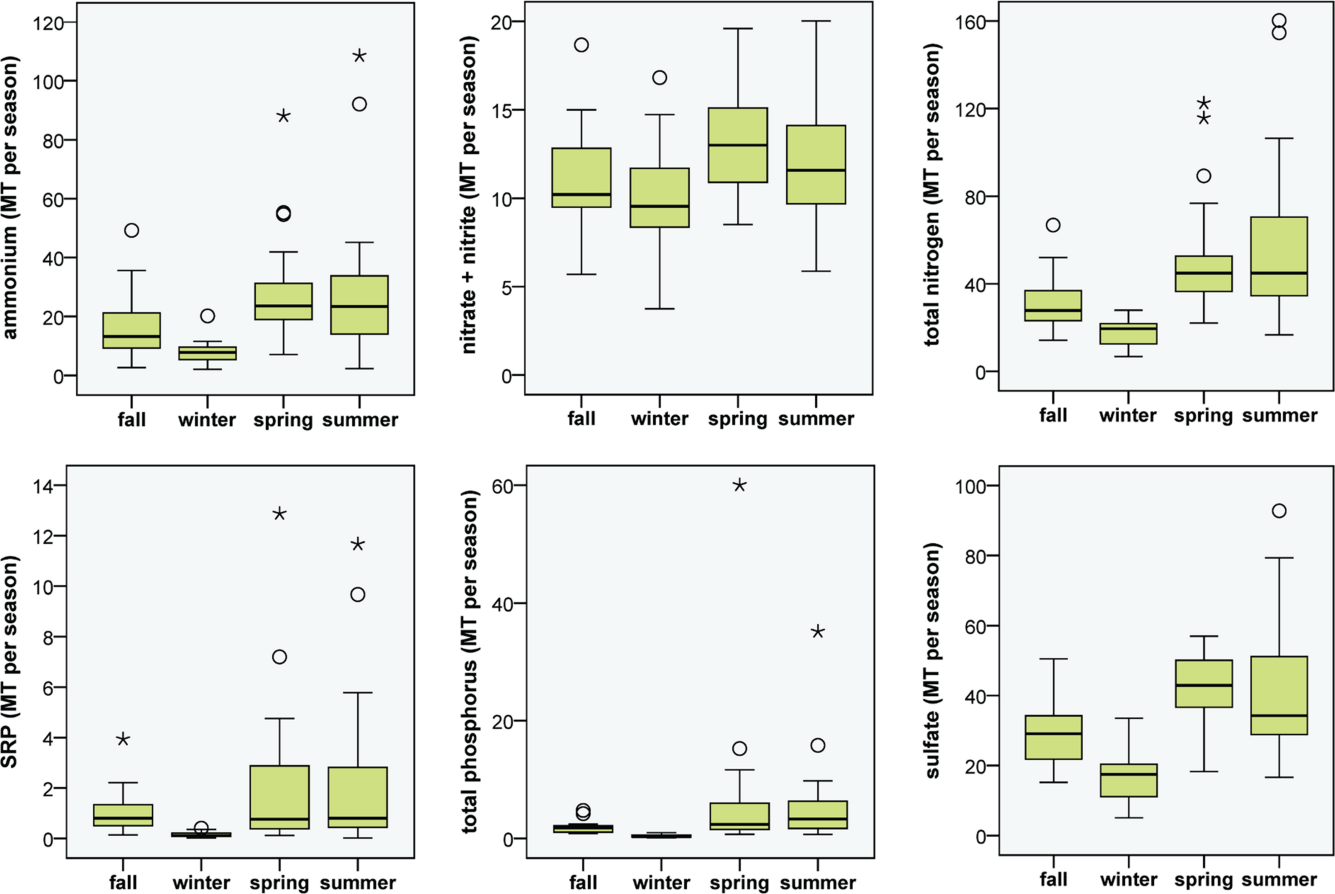
Seasonal atmospheric loading of N, P and SO_**4**_. Boxplots of seasonal N, P and SO_4_ loading to Flathead Lake from atmospheric deposition at the Flathead Lake Biological Station during the period 1982, 1985–2000. The box represents values for 50% of the cases with median displayed. Whiskers denote the range of values that were not outliers. Outliers are shown as circles and extreme values as asterisks.

**Figure 4:**
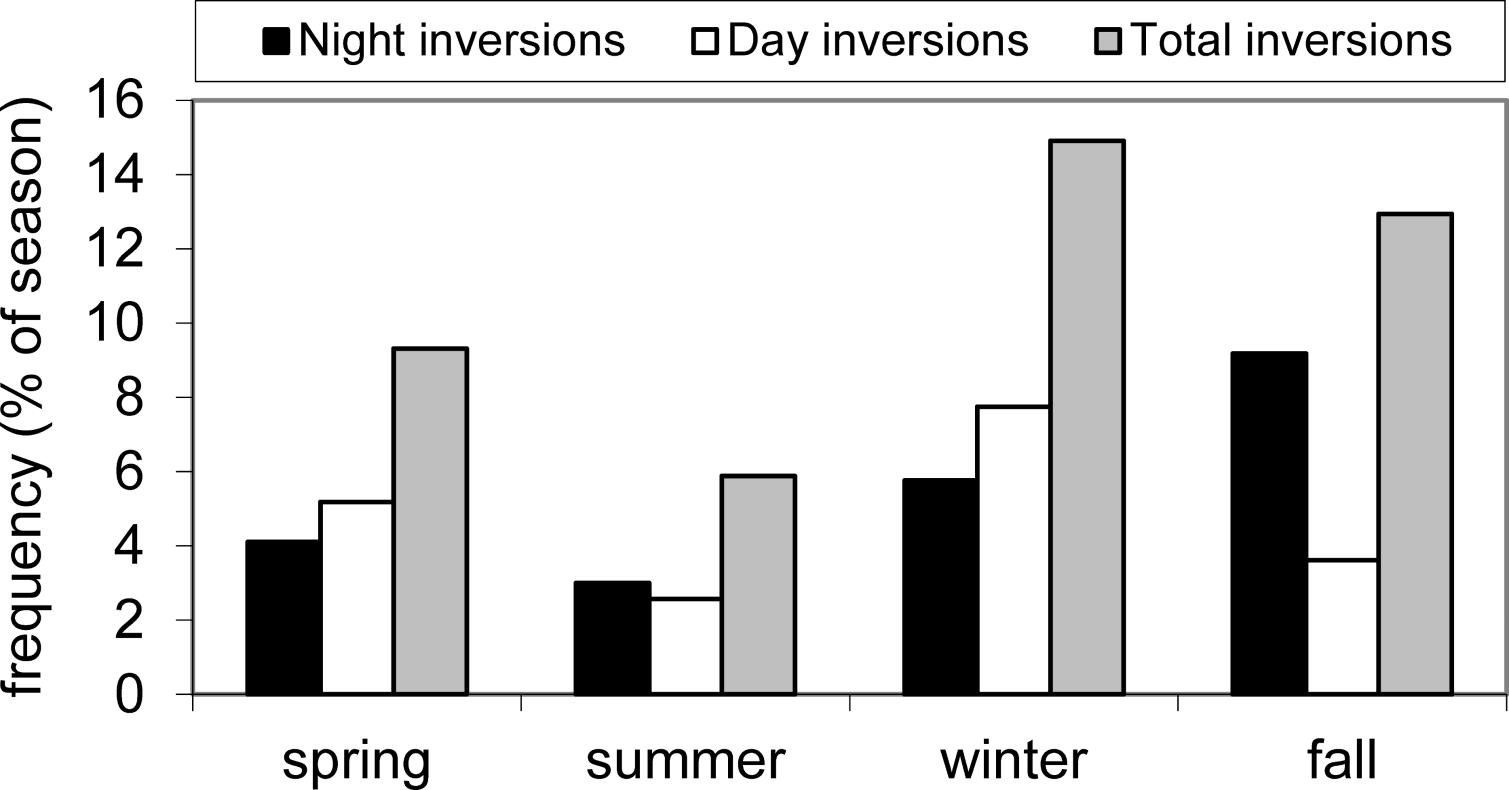
Frequency of air inversions. Average seasonal frequency of inversions derived from Noisy Basin, Montana SNOTEL and Flathead Lake Biological Station NOAA air temperature data. Data from 1994–2001.

In general, higher medians and a greater range of N loading estimates for atmospheric deposition were observed in spring and summer, though the range in NO_2/3_ loading was large for all seasons, and winter deposition of NH_4_ and TN was low most years (Fig. 3). The long-term upward trend in atmospheric deposition of NH_4_ was most evident in winter (Fig. S2), and it appeared that NO_2/3_ increased predominantly in winter (*p* value close to significance at 0.05), when air inversions persisted in the Flathead Valley (Fig. 4). There was some indication that loading of NO_2/3_ andNH_4_ also increased in the fall (i.e., *p* < 0.05 for 1981–2000 NO_2/3_; *p* = 0.06 for 1981–2004 NH_4_).

We observed significant positive correlations (*p* < 0.05) between our N loading estimates (i.e., NH_4_, NO_2/3_, TN) and the mass of fine aerosol particles of related species (i.e., NO_3_, NH_4_NO_3_, (NH_4_)_2_SO_4_) determined at the IMPROVE monitoring site (Table S4) suggesting a strong link among N deposition and local air quality. IMPROVE measures of fine soil mass also were positively correlated with both NH_4_ and TN loading indicating that at least some portion of the N loading was associated with fine soil dust.

Significant correlation of daily aerosol index (AI) and atmospheric loading of TN, NH_4_, NO_2/3_ (as well as P forms discussed below) was observed. AI exhibited a distinct cyclic annual pattern (Fig. S3); maximum positive AI values occurred in spring or late summer/early fall while maximum negative values were in winter, when air inversions were more frequent. Positive AI values represent UV-absorbing particles, such as dust and smoke while negative AI values frequently represent nonabsorbing aerosols, such as sulfates and hydrocarbons, mostly from industrial emissions with particle sizes near 0.1 to 0.2 µm or less (Herman et al., 1997). Mean monthly atmospheric loading of TN and NH_4_ were significantly correlated with mean monthly AI (*p* < 0.01), indicating that some of the load derived from sources outside the Flathead Basin as documented elsewhere (Herman et al., 1997). When examined seasonally, winter negative AI correlated with TN loads (*p* < 0.05) while summer average AI correlated with NO_2/3_ loads (*p* < 0.01). While the association with positive AI values could be explained by the apparent sources of N associated with dust and smoke, it is unclear as to the association with negative AI. IMPROVE data showed that the mass of fine aerosol particles of NH_4_NO_3_ and (NH_4_)_2_SO_4_ correlated with nutrient loading to the lake, thus it seems plausible that one or both of these aerosol constituents may be a source of the negative AI, as has been observed for H_2_SO_4_ (Torres et al., 1995).

USEPA point and area source emission data for the 11 counties surrounding Flathead Lake indicated that NO_x_ emissions decreased and NH_3_ emissions increased over the 1990–2000 period. This suggests that local sources contributed increasing NH_4_ loading to the lake, while more distant sources and/or fires (not included in the emission source data) increased NO_x_ loading.

Buildup of atmospheric particulates (PM10) coincided with local fire events (hectares burned) in many but not all years (Fig. S4). Significant correlations were found between hectares burned and mean PM10 concentrations at the Polson site in summer (*p* < 0.05) and the Kalispell site in fall (*p* < 0.01). NH_4_ and TN loads were frequently elevated when controlled slash and agricultural burns or forest fires occurred during inversions that trapped dense smoke in the Flathead valley. Severe air inversions occurred episodically year-round and increased the potential for substantial nutrient loading from even small local sources (e.g., NH_4_ loads were significantly correlated with hectares burned in spring, *p* < 0.01). As was observed for PM10 concentrations, there were also years when elevated N was unrelated to local fires (e.g., 1993) due to meteorological conditions that transported smoke out of the valley (Fig. S5). Despite the lack of coherence for each fire event, total hectares burned were significantly correlated with atmospheric TN loading (*p* < 0.05).

Agricultural sources (livestock production and fertilizer application) were 85–97% of the total NH_3_ emissions estimated by EPA for the 11 county region and of that, 73% was attributed to livestock production. One hotspot of NH_3_ emissions from livestock operations in Idaho was identified during an annual modeling study (Tonnesen et al., 2003; Fenn et al., 2003a). The site is 400 km southeast and upwind of Flathead Lake and thus may represent a more distant source. Farmlands dominate the valley bottom areas on both ends of Flathead Lake and N fertilizer use is routine; use of aqueous NH_3_ was common prior to 2001, while the much more volatile urea has been in use since then. Use of aqueous NH_3_ and urea for agricultural fertilization is a potential source of atmospheric N and has been implicated as one of the N pollution sources to Lake Tahoe (Jassby et al., 1994). Agricultural fertilization also increases microbial processing of fixed N, volatile NH_3_ soil concentrations, and ultimately increases N gas emissions from ground water and soils (Vitousek et al., 1997).

### Atmospheric phosphorus deposition and sources

Inorganic phosphorus (SRP) accounted for about 40% of TP (Table 1).When the time series P data were deseasonalized, we observed a gradually decreasing trend in concentration of TP (*p* < 0.01; Fig. S6). In contrast to N, the long-term trend in P was a significant decline (Fig. 2); atmospheric loading of TP decreased 135% from 1985 to 2004. No significant trend in SRP loads occurred. Seasonal analyses showed a significant decline in SRP and TP during summer (Fig. S2). A significant decline in winter for the period 1981–2000 was also observed for TP, but an elevated value in 2002 resulted in lack of significance for the 1981–2004 period.

Although P deposition declined, the atmosphere is clearly a source of P to the lake. Both TP and SRP loading were strongly correlated with fine soil concentrations in the atmosphere (Table S4) and loading of TP was correlated with mean PM10 concentrations during the fall and winter (*p* < 0.05). As with N, strong correlations between our P loading data and these various measures of air quality in the basin suggest that dust (fine soil) and smoke that build up in the atmosphere during periods of poor air circulation likely are the primary sources of P. Dust and fires have been documented as sources of atmospheric P deposition in other studies (e.g., Bullejos et al., 2010; Vicars, Sickman & Ziemann, 2010). Source apportionment studies conducted in the Flathead Valley showed that three of the seven major towns in Montana that were designated nonattainment by the State for ambient air quality standards for particulate matter (PM10) were located in the Flathead Valley just north of the lake (Erp, Ugorowski & Watne, 2002). Atmospheric loading of P was frequently elevated during periods when fires were present in the region and PM10 concentrations were significantly correlated with local fires. In addition, average monthly SRP and TP atmospheric loading to Flathead Lake was significantly correlated with an AI greater than zero (*p* < 0.01 and *p* < 0.05), indicative of UV-absorbing aerosols, such as dust and smoke. Morales-Baquero, Pullido-Villena & Reche (2006) reported a highly positive correlation between AI and particulate matter and total P linked to dry atmospheric deposition. Note that on 11 September 1988, the AI was high at 1.9. The source of the elevated AI was undoubtedly smoke transported into the valley from fires near and far. About 486,000 hectares of Yellowstone National Park burned that summer and fall while locally about 21,000 hectares burned in Glacier National Park and the surrounding area. Nonetheless, we observed an overall decline in P loading for which we have no concrete explanation except to note that fugitive dust may have declined in relation to land use trending from agriculture to ranchettes and paving of rural roads.

### Atmospheric sulfate deposition and sources

When the time series SO_4_ concentration data were deseasonalized, we observed a gradually decreasing trend (*p* < 0.05) over the period (Fig. S7). The long-term trend of SO_4_ loading also significantly declined (Fig. 2) by 39.1% over the study period. Seasonal patterns of SO_4_ loading were similar to NH_4_ and TN, with higher medians and ranges in spring and summer and lowest values in winter; but the long-term decrease in SO_4_ was apparent summer and winter (Fig. S2).

Sulfate concentrations in wet deposition decreased at almost every NADP site in the continental U. S. from 1985 to 2002 (Lehmann, Bowersox & Larson, 2005), as we also observed for Flathead Lake. At the NADP site in Glacier National Park north of Flathead Lake, the concentration of SO_4_ in wet deposition decreased by 47% from 1985 to 2002, and likewise, point source SO_2_ emissions in the Flathead region declined about 42% from 1990 to the 1996–2001 period for the 11 counties surrounding the lake (USEPA Emissions and Inventory and Analysis Group, North Carolina). The primary point sources of SO_2_ in Montana are coal and oil combustion, followed by refineries and smelters (Erp, Ugorowski & Watne, 2002), but these sites are predominately outside the Flathead Basin. The significant correlation between winter atmospheric loading of SO_4_ and AI < 0 (*p* < 0.05) indicates that distant sources may be important; results also suggest that this satellite-derived measure may be sensitive at fairly low levels of aerosol SO_2_. Negative AI values often represent the presence of nonabsorbing aerosols in the troposphere, usually from pollution in the form of sulfate aerosols (Herman et al., 1997).

As with N and P, SO_4_ loads strongly correlated with aerosol particle measures (winter PM10 concentrations, *p* < 0.05; fine soil, *p* < 0.05) and hectares burned (spring, *p* < 0.01) suggesting an association with smoke and dust entrainment. Maenhaut et al. (1996) found that prescribed fires gave rise to high levels of airborne soil dust and that sulfur was high in the fine fraction during the flaming phase, thus a correlation between SO_4_ and soil might be expected.

Finally, we expected an increase in pH in atmospheric deposition (Fig. S7; significant increase in pH at *p* < 0.05) due to decreased SO_4_ loading from sources near and far (i.e., thereby reducing inputs of sulfuric acid). However, NO_x_ emissions and NH_4_ and NO_3_ loading have increased. Elsewhere in the U.S., increases in NH_4_ and NO_3_ loading appear to be off-setting decreases in SO_4_ loading because they are precursors of nitric acid. Lake acidification may be linked to atmospheric N deposition in some areas (Vitousek et al., 1997) and may become a concern in poorly buffered lakes in our region, if N loading continues to increase.

### Comparison to other aquatic systems

Precipitation-weighted mean concentration of NO_2/3_ in atmospheric deposition was higher at Flathead Lake than both Swan (14 km E) and Whitefish (63 km NW) Lakes (Table S5); values at Whitefish were below the range of means determined for the entire period of record at Flathead Lake. TN in atmospheric deposition at Swan and Whitefish Lakes were similar to Flathead Lake, though TP was 2X and 7X that of Flathead, respectively. In contrast, values were considerably higher for Flathead Lake compared to data from the remote, high elevation site (57 km SE at Pendant Pass) for all variables; indeed, mean SRP was 21X higher, while TP and NH_4_ were 7–8X higher. Although the NADP site in Glacier National Park measured wet deposition only, mean concentrations of NH_4_ and NO_2/3_ at Flathead Lake were 5.6X and 15X higher, respectively. Mean sulfate concentrations at Flathead Lake were only somewhat higher (1.4–1.5X) than those measured at Pendant Pass and Glacier National Park. Lehmann, Bowersox & Larson (2005) showed the concentration of NH_4_ in wet deposition increased by 30% from 1985 to 2002 at the NADP site in Glacier National Park (GNP; see Fig. 1), but NO_2/3_ did not change. On the contrary, we observed an 84% increase in NO_2/3_ and a much larger increase in NH_4_ (198%) from 1985 to 2004 at Flathead Lake. Comparisons between the atmospheric collector at Flathead Lake and the NADP collector at GNP showed the precipitation-weighted mean concentration of NH_4_ at Flathead Lake was 5.6X that at GNP for the 1993–1994 fall to spring period (Table S5). These differences were due in part to the inclusion of dry deposition in the FLBS collector and the lack of dry deposition in the NADP collector. The propensity for inversions in the Flathead and differences in local sources likely also contributed to the observed differences.

Even accounting for potential dilution of ions from the greater precipitation at higher elevation, we still found substantially higher precipitation-weighted mean concentrations of NH_4_, SRP, and TP in the collector at FLBS on Flathead Lake compared to the collector at Pendant Pass in the Bob Marshall Wilderness, indicating the extent of anthropogenic N and P inputs. If all nutrient loading at Pendant Pass was from natural sources, even though there can be global movement of anthropogenic N and P even at high elevations (Holtgrieve et al., 2011), and we also account for dilution, then as much as 47% of NH_4_, 83% of SRP, and 43% of TP in atmospheric deposition at Flathead Lake during the 1993–1994 sampling period likely was anthropogenic in origin. It is also possible that inversions may contribute to increased atmospheric loading even under completely natural conditions.

The long-term range in inorganic N deposition (wet plus dry) to Flathead Lake was 3.0–13.0 g N ha^−1^ d^−1^, exceeding that reported by Bergstrom & Jansson (2006) for wet deposition in 1999 to other mountain lakes in the western U. S. (2.7–9.9 g N ha^−1^ d^−1^); the mean for Flathead was at the upper end of that range (7.6 g N ha^−1^ d^−1^). Summer loading of P was relatively high some years, but the 21-year mean for Flathead Lake was low (1.33 g P ha^−1^ d^−1^) in comparison to other lakes (Table S6). Jassby et al. (1994) reported a mean annual estimate of P deposition of 0.90 g P ha^−1^ d^−1^ for Lake Tahoe, California in 1989–1991. The long-term annual mean for Flathead Lake was similar at 0.84 ± 0.93 g P ha^−1^ d^−1^.

### Atmospheric versus tributary loading

Water flux through Flathead Lake is dominated by its largest tributary, the Flathead River. On average, 98% of the water contributed to the lake is from tributaries while only 2% is from precipitation. Thus, it was surprising to learn that NH_4_ loading from atmospheric deposition was almost on par with tributary loading: 44% of the total load (range 23–61%) was from the atmosphere (Table 2, Fig. S8). At a site near the Flathead River mouth, NH_4_ averaged 10 µg L^−1^ while the precipitation-weighted mean concentration of NH_4_in atmospheric deposition was 390 µg L^−1^. On the other hand, the contribution of NO_2/3_ from atmospheric sources to the lake was low (i.e., 6% of the total load), so tributary sources dominated the total NO_2/3_ load. Average annual NH_4_–N loading from atmospheric deposition was about twice that of NO_2/3_–N loading. Atmospheric deposition of NH_4_ also contributed the bulk of the N loading to Lake Tahoe (Dolislager et al., 2012), but unlike Flathead Lake, precipitation accounts for about half of the water entering Lake Tahoe.

**Table 2:** Atmospheric and tributary loading of N, P and **SO_4_** to Flathead Lake. Average daily loading of N, P and SO_4_ (g ha^−1^ d^−1^ ± 1 SD) to Flathead Lake from an atmospheric deposition collector for water years during the period 1982–2004 (excluding 1983 and 1984; NH_4_ and SO_4_ data unavailable in 1982) and from the tributaries for the same period (excluding 1983, 1984, 2001, 2002; TN, NH_4_ and SRP unavailable in 1982; NH_4_ unavailable 1992, 1993, 1998–2000). Tributary loads were apportioned relative to the lake surface area for comparison to deposition estimates. Bioavailable-P (BioP) in atmospheric deposition and tributary loads is also shown (see methods). Included are the dissolved inorganic and total molar N:P ratios.

	NH_4_–N	NO_2/3_–N	TN	SRP	TP	BioP	SO_4_	DIN:DIP	TN:TP
	(g ha^−1^ d^−1^ ± 1 SD)							(Molar ratio)	
Atmospheric									
Mean	4.9 ± 2.7	2.7 ± 0.5	9.2 ± 3.5	0.32 ± 0.28	0.8 ± 0.9	0.65 ± 0.71	7.5 ± 1.7	124 ± 126	39 ± 20
Range	1.6–12	1.7–3.6	5.7–18	0.03–0.89	0.2–4.2	0.12–3.2	4.8–12	14–492	8–89
*n* (years)	17	21	21	21	21	21	19	17	21
Tributaries									
Mean	6.3 ± 3.0	40 ± 13	102 ± 69	0.90 ± 0.35	21 ± 20	6.9 ± 3.2		113 ± 25	17 ± 9
Range	3.1–12	25–68	49–330	0.45–1.7	4.4–76	3.8–14		79–154	4–35
*n* (years)	11	19	18	18	19	19		11	18

Loading of biologically available P to Flathead Lake was dominated by tributary inputs, with total tributary loading about 10X that of atmospheric deposition. Inorganic phosphorus (SRP) was similar, contributing a much smaller proportion to TP in tributary loading than atmospheric loading to the lake (i.e., 4% versus 38%). Loading of organic N was dominated by tributary sources. Tributary loads of TN, NH_4_, and SRP declined 23% (*p* < 0.05), 41% (*p* < 0.001), and 63% (*p* < 0.0001), respectively.

**Figure 5:**
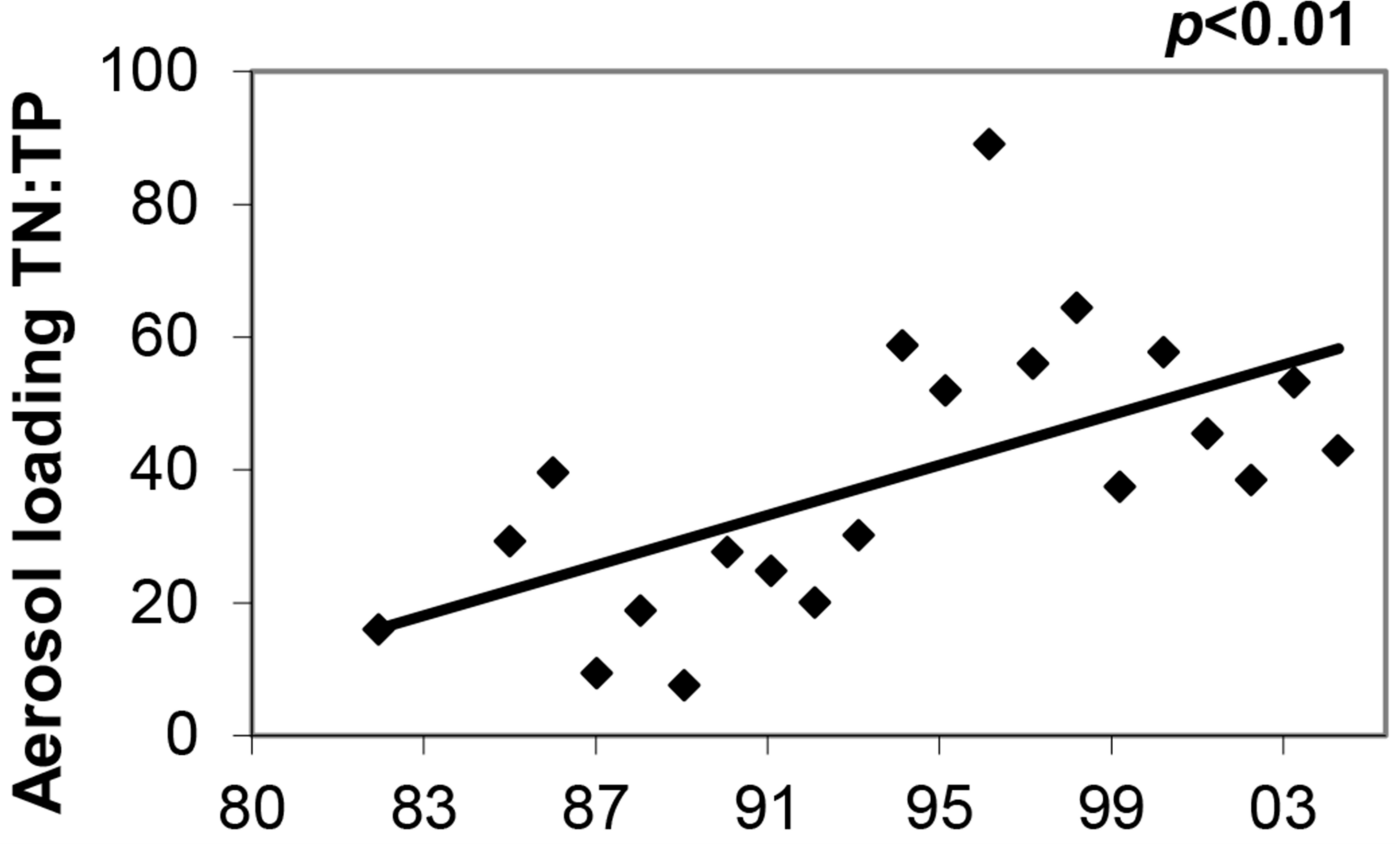
Molar TN:TP in atmospheric loading. Ratio of TN:TP (molar) in atmospheric deposition loading to Flathead Lake, 1982 and 1985–2004.

Although the proportion of total N and P that was inorganic was much greater in atmospheric loading than in tributary loading, the mean molar ratio of DIN:DIP was high and similar for both (i.e., 124 and 113, respectively; Table 2). But, DIN:BioP (14 ± 3; range 10–20) was an order of magnitude lower because most of the sediment-bound P that is discharged into the lake during spring snowmelt is biologically unavailable. The mean molar ratio of TN:TP for atmospheric deposition to Flathead Lake was 39, more than twice the ratio of 17 for tributary loading. The molar ratio of TN:TP in atmospheric deposition increased dramatically from 1982 to 2004 as a consequence of increasing N loading and decreasing P loading (Fig. 5). There was no significant trend in TN:TP for tributary loading.

### Importance of atmospheric loading to Flathead Lake

When mean NH_4_ plus NO_2/3_ loading from atmospheric deposition to Flathead Lake (1.31 × 10^8^ g N year^−1^) is converted to production based on a molar C:N ratio of 7 (Redfield, 1958), annual production from this source would be approximately 1.70 × 10^9^ g C year^−1^. The measured mean annual primary productivity of Flathead Lake was 4.63 × 10^10^ g C year^−1^ (i.e., 98 C m^−2^ year^−1^ converted for entire lake) during 1984–2004. Thus, an average of 3.7% of carbon production in Flathead Lake can be attributed to labile N inputs from atmospheric deposition, given that productivity is colimited by both N and P. Moreover, on years of high atmospheric inorganic N loading (e.g., 2002), deposition accounts for up to 6.9% of the organic carbon production in the lake.

Nutrient bioassay studies conducted in the late 1980s showed Flathead Lake was colimited by both N and P (Spencer & Ellis, 1998). The concentration of NO_2/3_ in the epilimnion drops from about 60 µg L^−1^ before stratification to below detection limits (i.e., <0.6 µg L^−1^) by late summer, while NH_4_ remains below the detection limit almost year-round. During mid- to late stratification (summer and fall) when both inorganic forms of N are below detection limit, atmospheric inputs, high in inorganic N, may have the potential to increase production in the euphotic zone. Unlike tributary inputs, the great majority of which are delivered at the north end of the lake during spring freshet, precipitation occurs lake wide directly to the lake surface and photic zone. We hypothesize that the total load and timing of this atmospheric pulsing of N may be an important mechanism regulating lake productivity.

Atmospheric deposition is relatively depleted in P and Si (Duce et al., 1991 and depleted P in this study), thus alteration of stoichiometric ratios of N:P:Si could potentially influence phytoplankton community composition. Numerous studies in coastal waters and seas have described shifts in dominant phytoplankton species, including bloom species, as a result of declining Si:N or Si:P loading (Paerl, 1997). Exogenous N and other limiting nutrient inputs are thought to play a critical role in the development of harmful algal blooms (Paerl, 1997). In contrast, in Flathead Lake, blooms of the noxious blue–green algae *Anabaena flos-aquae* (able to fix N from the atmosphere) have disappeared in recent years as N loading has increased. Additional research is clearly warranted. Bergstrom & Jansson (2006) and Elser et al. (2009) report that increased deposition of inorganic N over large areas of Europe and North America has caused elevated concentrations of inorganic N in lakes and has probably caused a shift from natural N limitation to P limitation in many unproductive lakes. Increasing N and N:P imbalances in lakes may result in reduced biodiversity and reduced production of higher trophic levels (Elser et al., 2009). Finlay, Small & Sterner (2013) suggest that in P- and carbon-poor lakes receiving excess N (such as Flathead Lake) that water column N accumulation and downstream flushing result, because these lakes lack an efficient mechanism to remove N. Our long-term data support their findings; N is increasing in the lake and in the outlet and thus increased downstream transport of N from Flathead Lake should clearly be a management concern.

## Conclusions

This study contributes to our understanding of the importance of atmospheric deposition to lake nutrient budgets, the potential impact of air inversions and the role fire may play in N and P deposition in this western region of the U.S. From 1985 to 2004, NO_2/3_ and NH_4_ loading increased by 48 and 198% and total P and SO_4_ decreased by 135 and 39%. Severe air inversions occurred periodically throughout the year and increased the potential for substantial nutrient loading and buildup of particulates from local sources (e.g., biomass burning in spring). A comparison between atmospheric loading of nutrients at a high elevation wilderness site to that at Flathead Lake indicated that inversions in the Flathead Valley greatly increased the concentration of the measured variables. Numerous measures of air quality in the basin were correlated with our N, P, and SO_4_ loading data suggesting that both dust and smoke are important sources. Point and area source emission data indicated that local emission sources are likely contributing to increased NH_4_ loading to the lake, but more distant sources and/or fires may be important NO_x_ sources. The decreasing trend in SO_4_ load reflected the decline in point source SO_2_ emissions in the region, but significant correlations between winter atmospheric loading of SO_4_ and AI < 0 indicated distant sources also may be important. This study also showed that atmospheric deposition is a major contributor of NH_4_ to the lake despite the fact that tributaries account for 98% of the water budget. Atmospheric deposition of NH_4_ was on par with that from the tributaries, averaging 44% of the total load and on some years exceeding tributary loading. Thus, aerosol deposition is an important water quality concern at Flathead Lake and likely also for other lakes in the region.

## Supplemental Information

10.7717/peerj.841/supp-1 Supplemental Information 1 Atmospheric nutrient and sulfate deposition data and various measures of air quality This data file includes the following: nutrient and sulfate concentrations in atmospheric deposition at the Flathead Lake Biological Station (FLBS) and two other lake basins in the region, precipitation volume at the NOAA site at the FLBS, principal fine aerosol species data from the IMPROVE FLAT1 site, wildfire start dates and hectares burned on Flathead National Forest and Montana Department of Natural Resources and Conservation lands, aerosol particulate matter <10 um concentrations for Kalispell, Montana, daily aerosol index values for the region centered over Flathead Lake, and frequency of air inversions in the Flathead Lake basin.

10.7717/peerj.841/supp-2 Table S1 Morphometric, hydrologic and limnological parameters for Flathead Lake, Montana (Fig. 1) Morphometric features were based on measurements at a lake elevation of 879 m above mean sea level. Monthly or more frequent measures of limnological parameters were made from 1988 to 2004 at the 116 m deep long-term monitoring site 1.5 km west of FLBS. Mean annual chlorophyll a, nitrogen and phosphorus measures were determined for 0–30 m photic zone samples collected using an integrating hose. Primary productivity was measured at six depths throughout the photic zone using in situ 14C uptake in light/dark bottle experiments. See Ellis et al. (2011) for additional methods.

10.7717/peerj.841/supp-3 Table S2 Comparison of two methods for collection of dry deposition Mean deposition of N, P and SO_4_ in g ha^−1^ day^−1^ for 2 types of collectors during very smoky atmospheric conditions in 2003 and during clear conditions in 2004 at the FLBS (*n* = 2). The DI collectors contained ultrapure distilled water. The dry deposition collectors were identical but did not contain water.

10.7717/peerj.841/supp-4 Table S3 Methods used in monitoring water quality in atmospheric deposition Methods used in monitoring water quality in atmospheric deposition (wet plus dry deposition) collected on the weather tower on Yellow Bay Point of Flathead Lake, Montana.

10.7717/peerj.841/supp-5 Table S4 Significant regressions of atmospheric loading and IMPROVE measures *P* values from significant regressions of FLBS atmospheric deposition loading (MT month^−1^) against the monthly mean mass of fine aerosol particles <2.5 µm in diameter (µg m^−3^) and coarse mass of particles 2.5–10 µm in diameter (µg m^−3^) for the period June 2002–December 2004. The principal fine aerosol species data are from the IMPROVE site near the city of Polson ( http://vista.cira.colostate.edu/improve; see Fig. 1). All were positive correlations except TP versus fine NO_3_ and TP versus fine NH_4_NO_3_.

10.7717/peerj.841/supp-6 Table S5 Regional means of N, P and SO_4_ concentrations in atmospheric deposition Precipitation-weighted means of chemical data from atmospheric deposition collectors located on Flathead Lake, Swan Lake, Whitefish Lake and Pendant Pass in the Bob Marshall Wilderness and from the National Atmospheric Deposition Program (NADP) wet deposition collector at West Glacier, Montana. Data are means from the fall to spring period, to allow comparisons to results from the Pendant Pass collector. Site elevation and distance and direction from Flathead Lake are also presented.

10.7717/peerj.841/supp-7 Table S6 Atmospheric deposition of P (g ha^−1^ d^−1^) in summer for various lakes Estimates of atmospheric deposition of P (g ha^−1^ d^−1^) in summer for various lakes. The range for Flathead Lake represents data from 21 summers during the period 1981–2004. Swan Lake atmospheric deposition samples were collected in 1993. Summer 1983 estimates for the north and south atmospheric deposition collectors on Whitefish Lake are presented.

10.7717/peerj.841/supp-8 Figure S1 Annual and mean monthly precipitation at FLBS, 1939–2004 Total annual and mean monthly precipitation (cm) at the NOAA site at the Flathead Lake Biological Station, east shore of Flathead Lake, during the period of this study. Dotted line in top panel is the long-term mean (1939–2004) and whiskers in bottom panel denote +1.0 standard deviation.

10.7717/peerj.841/supp-9 Figure S2 Significant trends in seasonal atmospheric loading of N, P and SO_4_ Significant trends in the seasonal loading of N, P, and SO_4_ to Flathead Lake from atmospheric deposition. Total seasonal load is shown in metric tons (MT) with best-fit linear regression.

10.7717/peerj.841/supp-10 Figure S3 Daily aerosol index and aerosol index during temperature inversions Daily AI for the 50 km × 50 km region centered over the midlake deep site in Flathead Lake (i.e., 2 km west of Yellow Bay Point), 1981–2001 and AI during temperature inversions at Flathead Lake, Montana, 1996–2000.

10.7717/peerj.841/supp-11 Figure S4 Hectares burned on forest lands and PM10 concentrations Wildfire start dates and hectares burned on Flathead National Forest and Montana Department of Natural Resources and Conservation lands and daily mean PM10 concentrations (µg m^−3^ of aerosol particles <10 µm in diameter) for Kalispell, Montana. Tick marks on *x*-axis represent January 1 of each year. *Y*-axis for hectares burned expanded to show smaller fire events.

10.7717/peerj.841/supp-12 Figure S5 Hectares burned on forest lands and atmospheric loading of total N and P Wildfire start dates and hectares burned on Flathead National Forest and Montana Department of Natural Resources and Conservation lands and FLBS atmospheric loading of TN and TP (MT day^−1^) are presented. *Y*-axis for hectares burned expanded to show smaller fire events.

10.7717/peerj.841/supp-13 Figure S6 Trends in NH_4_, NO_2/3_ and total N concentrations in atmospheric deposition The natural log of nitrogen concentration (µg L^−1^) in atmospheric deposition at the Flathead Lake Biological Station and the best-fit seasonal trend are shown in the graphs at left. A least squares regression of the deseasonalized data (residuals) against date display the linear trend in the graphs at right.

10.7717/peerj.841/supp-14 Figure S7 Trends in total P and SO_4_ concentrations and pH in atmospheric deposition The natural log of TP and SO_4_ concentrations (µg L^−1^ and mg L^−1^, respectively) and pH values in atmospheric deposition at the Flathead Lake Biological Station and the best-fit seasonal trend are shown in the graphs at left. A least squares regression of the deseasonalized data (residuals) against date display the linear trend in the graphs at right.

10.7717/peerj.841/supp-15 Figure S8 Total nutrient load to Flathead Lake Discharge of the major tributaries to Flathead Lake and precipitation volume at the NOAA site at the Flathead Lake Biological Station by water year are shown in the top left graph. Total nutrient load to the lake from atmospheric deposition (dark portion of histogram) and tributary plus septic system and sewage treatment plant sources (light portion of histogram) are shown in the remaining graphs. Asterisks denote lack of tributary loads for water years when atmospheric loading was determined. Estimates of nutrient loading from septic systems were from Makepeace & Mladenich (1996). Nutrient loading from sewage treatment plants were calculated from daily discharge measurements and monthly nutrient concentrations obtained from Montana Department of Environmental Quality.
