# Supplementary material for: Long-term atmospheric deposition of nitrogen, phosphorus and sulfate in a large oligotrophic lake
Source: PeerJ. 2015 Mar 19;3:e841. doi: 10.7717/peerj.841 (PMC4369344; doi:10.7717/peerj.841)
Supplement: Table S1 — Morphometric features were based on measurements at a lake elevation of 879 m above mean sea level. Monthly or more frequent measures of limnological parameters were made from 1988 to 2004 at the 116 m deep long-term monitoring site 1.5 km west of FLBS. Mean annual chlorophyll a, nitrogen and phosphorus measures were determined for 0–30 m photic zone samples collected using an integrating hose. Primary productivity was measured at six depths throughout the photic zone using in situ 14C uptake in light/dark bottle experiments. See Ellis et al. (2011) for additional methods. [file peerj-03-841-s002.docx]

| Maximum length | 43.9 km |
| --- | --- |
| Maximum width | 24.7 km |
| Shoreline length with islands | 267.0 km |
| Surface area | 500.2 km^2^ |
| Maximum depth | 116 m |
| Mean depth | 43.6 m |
| Volume | 21.8 km^3^ |
| Hydraulic retention time | 2.2 yr |
| Mean Secchi depth | 11.0 m |
| Primary productivity | 98 g C m^-2^ yr^-1^ |
| Chlorophyll *a* | 0.90 µg L^-1^ |
| Nitrate nitrogen | 33 µg N L^-1^ |
| Ammonium nitrogen | <5.5 µg N L^-1^ |
| Total nitrogen | 101 µg N L^-1^ |
| Soluble reactive phosphorus | <0.70 µg P L^-1^ |
| Total phosphorus | 5.5 µg P L^-1^ |
