# Supplementary material for: Long-term atmospheric deposition of nitrogen, phosphorus and sulfate in a large oligotrophic lake
Source: PeerJ. 2015 Mar 19;3:e841. doi: 10.7717/peerj.841 (PMC4369344; doi:10.7717/peerj.841)
Supplement: Table S2 — Mean deposition of N, P and SO4 in g ha−1 day−1 for 2 types of collectors during very smoky atmospheric conditions in 2003 and during clear conditions in 2004 at the FLBS (n = 2). The DI collectors contained ultrapure distilled water. The dry deposition collectors were identical but did not contain water. [file peerj-03-841-s003.docx]

|  | 2003 – Smoky | |  | 2004 – Clear | |
| --- | --- | --- | --- | --- | --- |
|  | DI collector  Aug 11–14 | Dry collector  Aug 11–14 |  | DI collector  Jun 1–5 | Dry collector  Jun 1–5 |
| NH_4_-N | 17.6 | 0.4 |  | 1.2 | 0.3 |
| NO_2/3_-N | 1.0 | 0.3 |  | 0.1 | 0.03 |
| TN | 19.0 | 2.9 |  | 8.8 | 8.4 |
| SRP | 0.5 | 0.3 |  | 0.2 | 0.2 |
| TP | 0.7 | 0.5 |  | 1.2 | 1.2 |
| SO_4_ | 2.7 | 0.9 |  | 1.2 | 1.2 |
