# Supplementary material for: Long-term atmospheric deposition of nitrogen, phosphorus and sulfate in a large oligotrophic lake
Source: PeerJ. 2015 Mar 19;3:e841. doi: 10.7717/peerj.841 (PMC4369344; doi:10.7717/peerj.841)
Supplement: Table S3 — Methods used in monitoring water quality in atmospheric deposition (wet plus dry deposition) collected on the weather tower on Yellow Bay Point of Flathead Lake, Montana. [file peerj-03-841-s004.docx]

| Variables | Method (references) | Detection limit |
| --- | --- | --- |
| Phosphorus |  |  |
| Total (TP) | Persulfate digestion; modified  automated ascorbic acid^a^ | 0.4 µg L^-1^-P |
| Soluble reactive (SRP) | Filtration; modified automated  ascorbic acid^a^ | 0.7 µg L^-1^-P |
| Nitrogen |  |  |
| Total (TN) | Persulfate digestion^b^; automated  cadmium reduction^a^ | 20.0 µg L^-1^-N |
| Nitrite + nitrate (NO_2/3_) | Automated cadmium reduction^a^ | 0.6 µg L^-1^-N |
| Ammonium (NH_4_) | Automated phenate^a^ | 5.5 µg L^-1^-N |
| Sulfate (SO_4_) | Ion chromatography^a^ | 0.05 mg L^-1^-SO_4_ |
| pH (units) | Electrode (low ion) | 0.1 units |

^a^ APHA (2005)

^b^ D'Elia et al. (1977)

References:

**American Public Health Association.** 2005. *Standard methods for the examination of water and wastewater*. District of Columbia, Washington: American Public Health Association.

**D'Elia CF, Steudler PA, Corwin N.** 1977. Determination of total nitrogen in aqueous samples using persulfate digestion. *Limnology and Oceanography* **22**:760–764.
