# Supplementary material for: Long-term atmospheric deposition of nitrogen, phosphorus and sulfate in a large oligotrophic lake
Source: PeerJ. 2015 Mar 19;3:e841. doi: 10.7717/peerj.841 (PMC4369344; doi:10.7717/peerj.841)
Supplement: Table S4 — P values from significant regressions of FLBS atmospheric deposition loading (MT month−1) against the monthly mean mass of fine aerosol particles <2.5 µm in diameter (µg m−3) and coarse mass of particles 2.5–10 µm in diameter (µg m−3) for the period June 2002–December 2004. The principal fine aerosol species data are from the IMPROVE site near the city of Polson ( http://vista.cira.colostate.edu/improve; see Fig. 1). All were positive correlations except TP versus fine NO3 and TP versus fine NH4NO3. [file peerj-03-841-s005.docx]

|  | Mass of aerosol particles (IMPROVE) | | | | | | |
| --- | --- | --- | --- | --- | --- | --- | --- |
| Aerosol  loading  (FLBS) | Fine  NO_3_ | Fine  NH_4_NO_3_ | Fine  SO_4_ | Fine  NH_4_SO_4_ | Fine  elemental C | Fine  soil | Coarse  mass |
| NH_4_-N | 0.04 | 0.04 | 0.05 | 0.02 |  | 0.006 | 0.05 |
| NO_3_-N | 0.03 | 0.03 |  |  | 0.03 |  |  |
| TN | 0.03 | 0.03 | 0.01 | 0.009 |  | 0.01 |  |
| SRP | 0.03 | 0.03 |  |  |  | 0.003 | 0.03 |
| TP | 0.008 | 0.008 |  |  |  | 0.006 |  |
| SO_4_ |  |  | 0.001 | 0.001 |  | 0.04 |  |
