# Supplementary material for: Long-term atmospheric deposition of nitrogen, phosphorus and sulfate in a large oligotrophic lake
Source: PeerJ. 2015 Mar 19;3:e841. doi: 10.7717/peerj.841 (PMC4369344; doi:10.7717/peerj.841)
Supplement: Table S5 — Precipitation-weighted means of chemical data from atmospheric deposition collectors located on Flathead Lake, Swan Lake, Whitefish Lake and Pendant Pass in the Bob Marshall Wilderness and from the National Atmospheric Deposition Program (NADP) wet deposition collector at West Glacier, Montana. Data are means from the fall to spring period, to allow comparisons to results from the Pendant Pass collector. Site elevation and distance and direction from Flathead Lake are also presented. [file peerj-03-841-s006.docx]

|  | 1982–1983 |  | 1992–1993 | |  | 1993–1994 | | |
| --- | --- | --- | --- | --- | --- | --- | --- | --- |
|  | Whitefish  Lake |  | Swan  Lake | Flathead Lake |  | Flathead Lake | Pendant Pass | NADP W. Glacier |
| Distance (km)/direction | 63NW |  | 14E |  |  |  | 57SE | 71N |
| Elevation (m) | 913 |  | 934 | 882 |  | 882 | 2042 | 980 |
| Variable (units) |  |  |  |  |  |  |  |  |
| NH_4_ (µg L^-1^-N) |  |  |  |  |  | 489 | 63 | 88 |
| NO_2/3_ (µg L^-1^-N) | 75 |  | 138 | 166 |  | 252 | 79 | 116 |
| TN (µg L^-1^-N) | 458 |  | 466 | 424 |  | 737 | 164 |  |
| SRP (µg L^-1^-P) |  |  |  |  |  | 14.5 | 0.7 |  |
| TP (µg L^-1^-P) | 105 |  | 26 | 15 |  | 23 | 3 |  |
| SO_4_ (mg L^-1^-SO_4_) |  |  |  |  |  | 0.49 | 0.31 | 0.36 |
