# Supplementary material for: Long-term atmospheric deposition of nitrogen, phosphorus and sulfate in a large oligotrophic lake
Source: PeerJ. 2015 Mar 19;3:e841. doi: 10.7717/peerj.841 (PMC4369344; doi:10.7717/peerj.841)
Supplement: Table S6 — Estimates of atmospheric deposition of P (g ha−1 d−1) in summer for various lakes. The range for Flathead Lake represents data from 21 summers during the period 1981–2004. Swan Lake atmospheric deposition samples were collected in 1993. Summer 1983 estimates for the north and south atmospheric deposition collectors on Whitefish Lake are presented. [file peerj-03-841-s007.docx]

| Lake | P Deposition | Study reference |
| --- | --- | --- |
| Lake Warniak, Poland | 6 | *Kowalczewski and Rybak, (1981)* |
| Como Creek, Colorado | 3–7 | *Lewis et al., (1985)* |
| Lake Dillon, Colorado | 22 | *Lewis et al., (1985)* |
| Piburger See, Austria | 2 | *Psenner, (1984)* |
| Mirror Lake, New Hampshire | 3–4 | *Cole et al., (1990)* |
| Flathead Lake, Montana | 0.3–8 | this study |
| Swan Lake, Montana | 3 | unpublished |
| Whitefish Lake, Montana | 1–2 | unpublished |

References:

**Cole JJ, Caraco NF, Likens GE.** 1990. Short-range atmospheric transport: A significant source of phosphorus to an oligotrophic lake. *Limnology and Oceanography* **35**:1230–1237.

**Kowalczewski A, Rybak JI.** 1981. Atmospheric fallout as a source of phosphorus for Lake Warniak. *Ekolgia Polska* **29**:63–71.

**Lewis Jr WM, Grant MC, Hamilton SK.** 1985. Evidence that filterable phosphorus is a significant atmospheric link in the phosphorus cycle. *Oikos* **45**:428–432.

**Psenner R.** 1984. The proportion of epineuston and total atmospheric inputs of carbon, nitrogen and phosphorus in the nutrient budget of a small mesotrophic lake (Piburger See, Austria). *Internationale Revue der Gesamten Hydrobiologie* **69**:23–39.
