# Supplementary figures and images for: Long-term atmospheric deposition of nitrogen, phosphorus and sulfate in a large oligotrophic lake
Source: PeerJ. 2015 Mar 19;3:e841. doi: 10.7717/peerj.841 (PMC4369344; doi:10.7717/peerj.841)

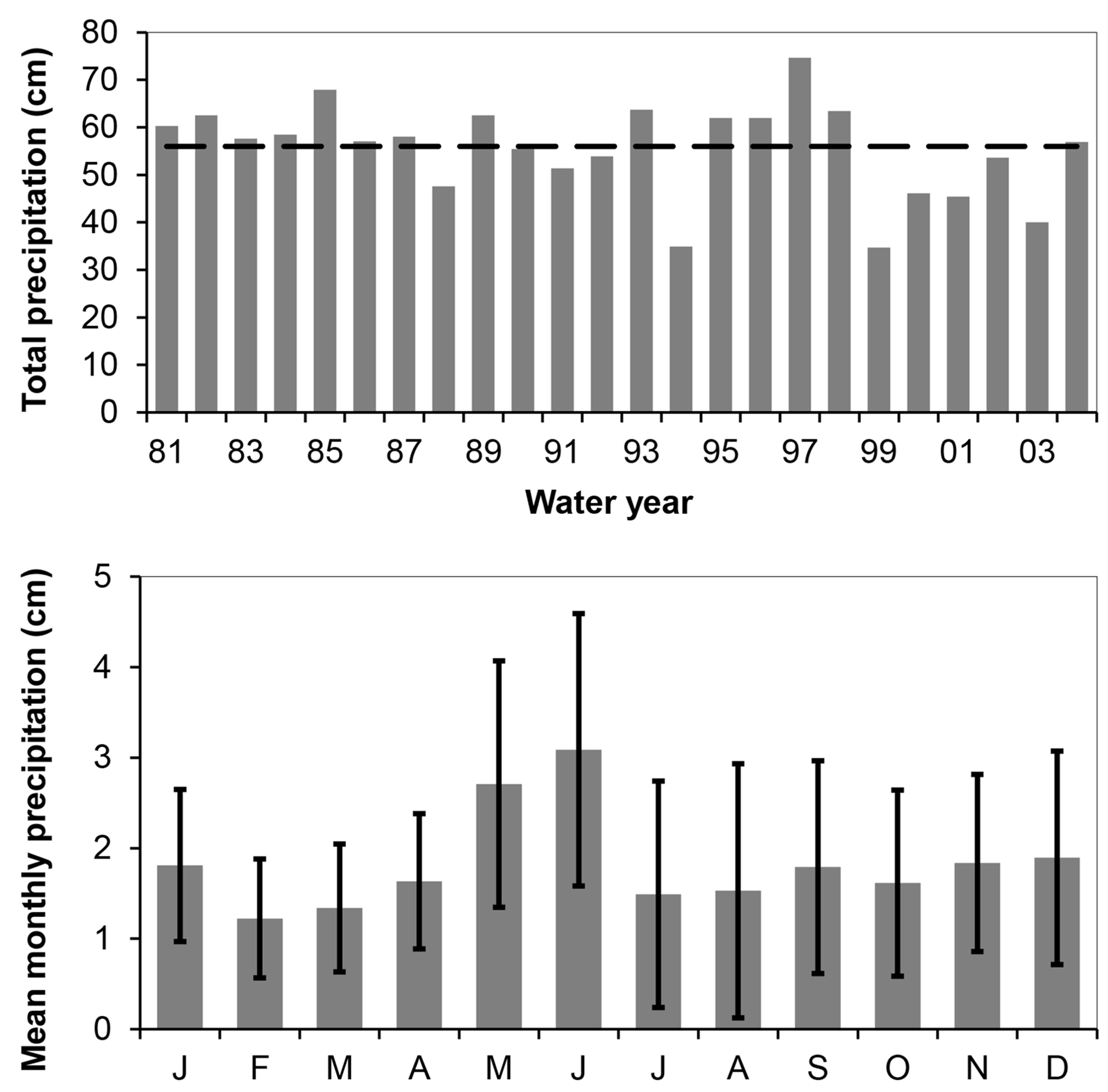

Supplement: Figure S1 — Total annual and mean monthly precipitation (cm) at the NOAA site at the Flathead Lake Biological Station, east shore of Flathead Lake, during the period of this study. Dotted line in top panel is the long-term mean (1939–2004) and whiskers in bottom panel denote +1.0 standard deviation. [file peerj-03-841-s008.png]

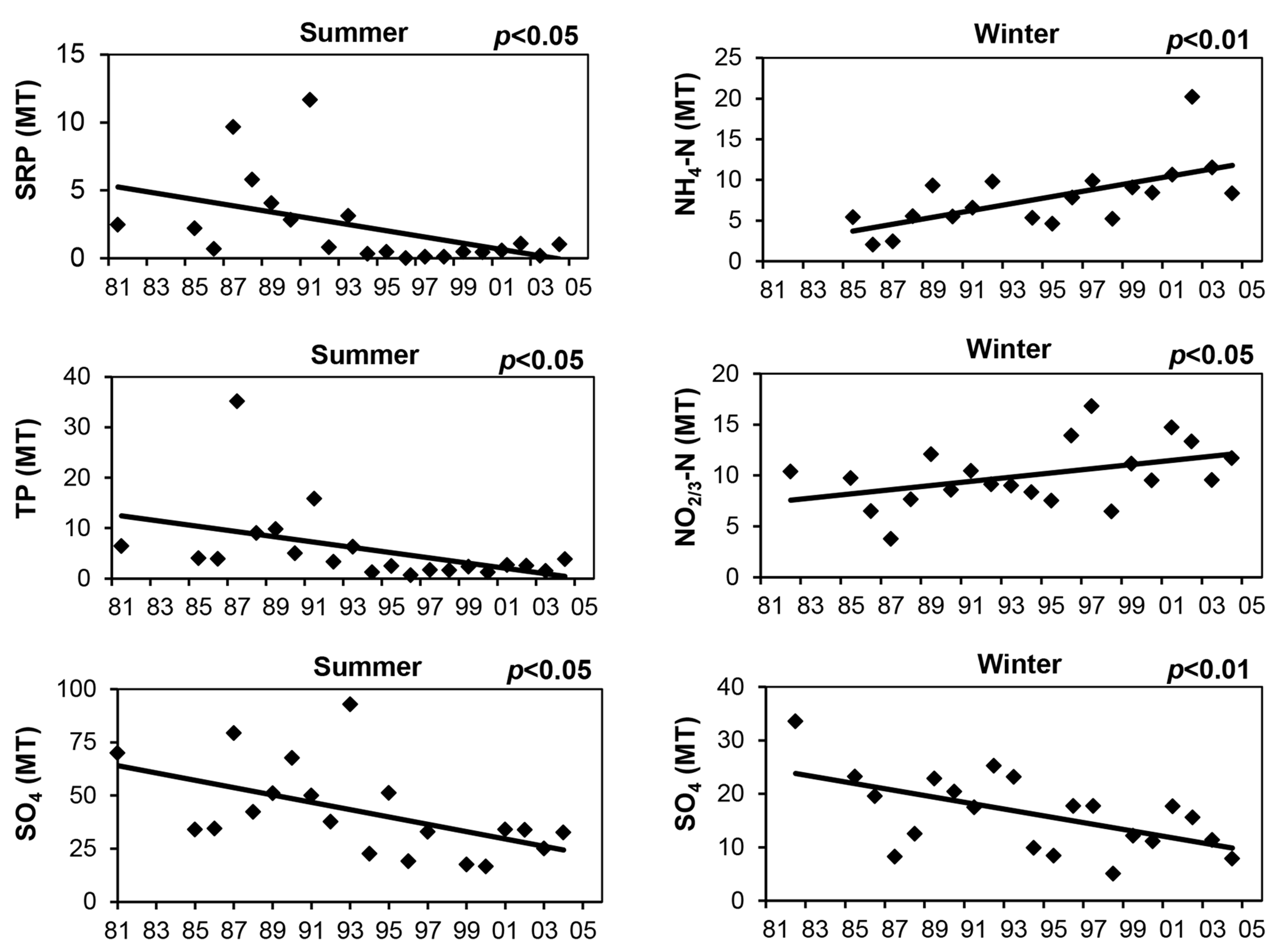

Supplement: Figure S2 — Significant trends in the seasonal loading of N, P, and SO4 to Flathead Lake from atmospheric deposition. Total seasonal load is shown in metric tons (MT) with best-fit linear regression. [file peerj-03-841-s009.png]

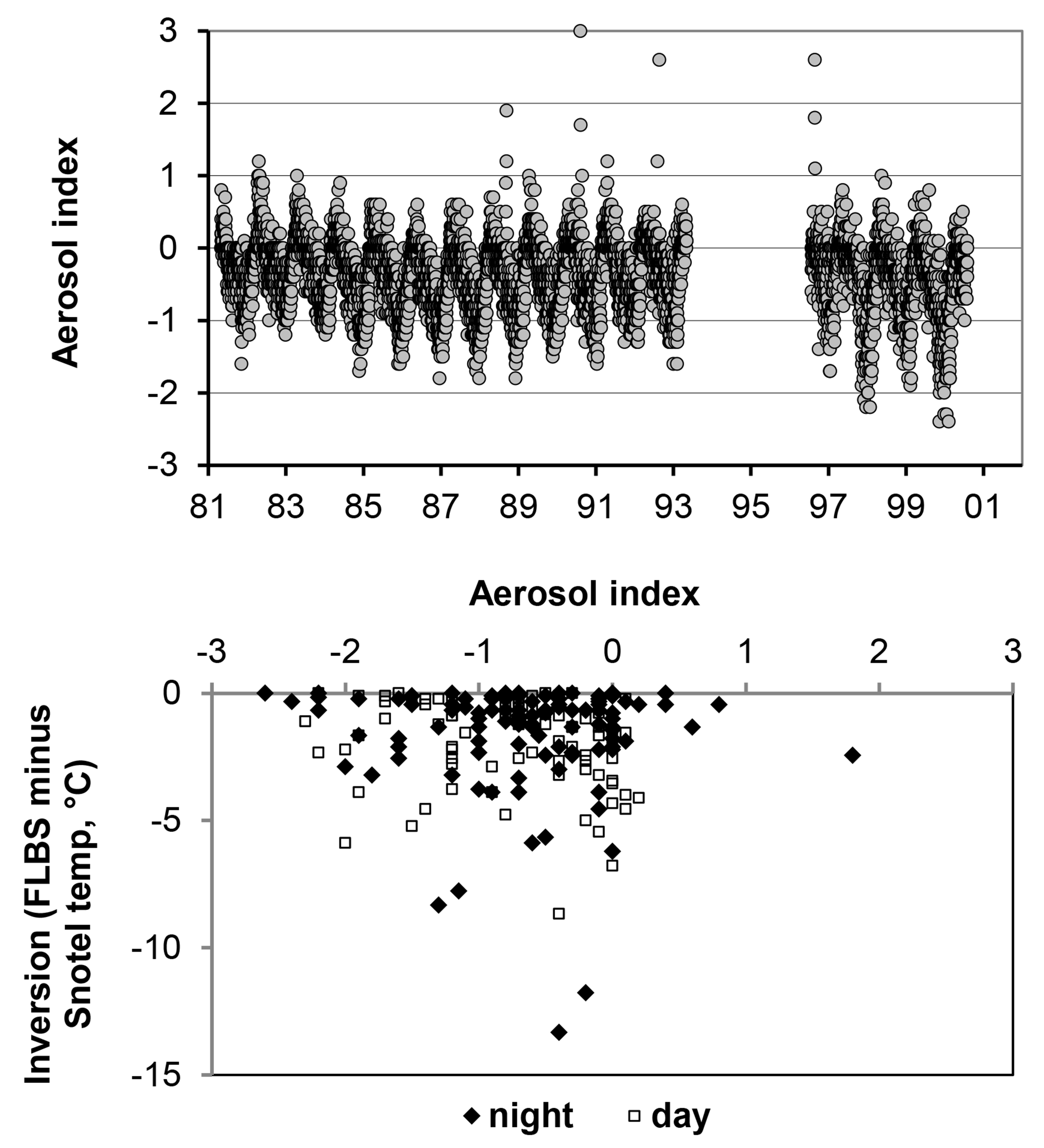

Supplement: Figure S3 — Daily AI for the 50 km × 50 km region centered over the midlake deep site in Flathead Lake (i.e., 2 km west of Yellow Bay Point), 1981–2001 and AI during temperature inversions at Flathead Lake, Montana, 1996–2000. [file peerj-03-841-s010.png]

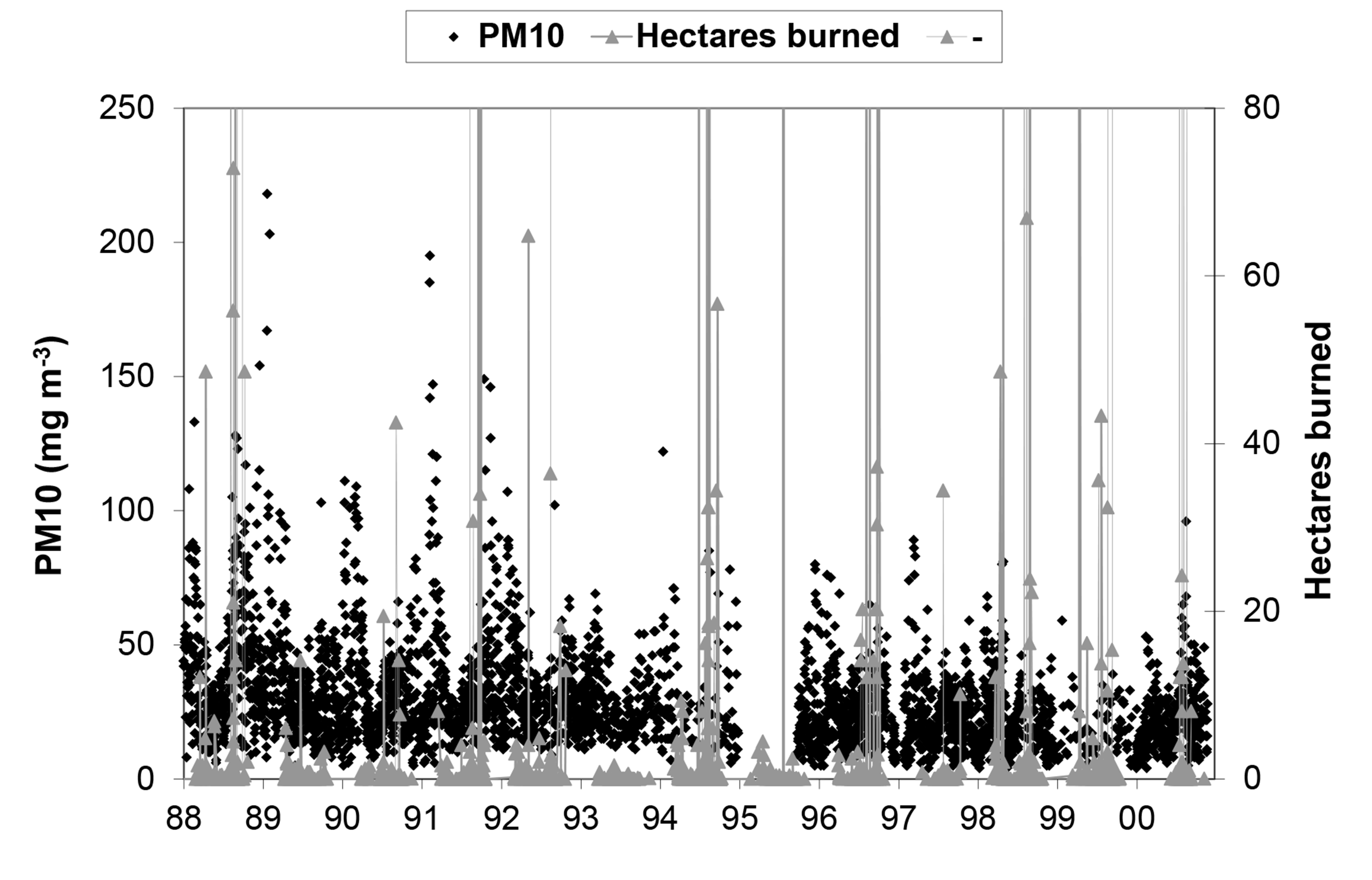

Supplement: Figure S4 — Wildfire start dates and hectares burned on Flathead National Forest and Montana Department of Natural Resources and Conservation lands and daily mean PM10 concentrations (µg m−3 of aerosol particles <10 µm in diameter) for Kalispell, Montana. Tick marks on x-axis represent January 1 of each year. Y-axis for hectares burned expanded to show smaller fire events. [file peerj-03-841-s011.png]

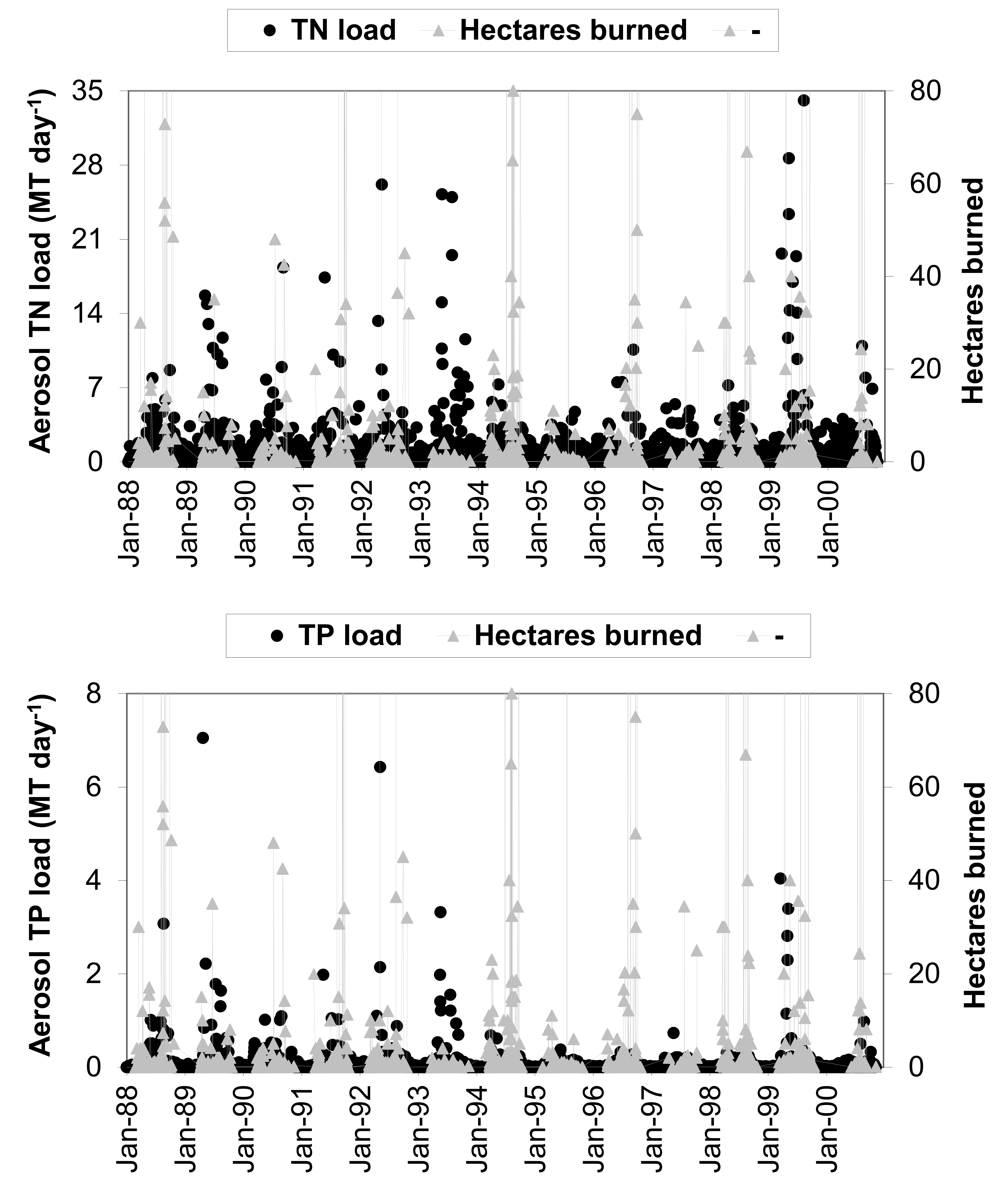

Supplement: Figure S5 — Wildfire start dates and hectares burned on Flathead National Forest and Montana Department of Natural Resources and Conservation lands and FLBS atmospheric loading of TN and TP (MT day−1) are presented. Y-axis for hectares burned expanded to show smaller fire events. [file peerj-03-841-s012.png]

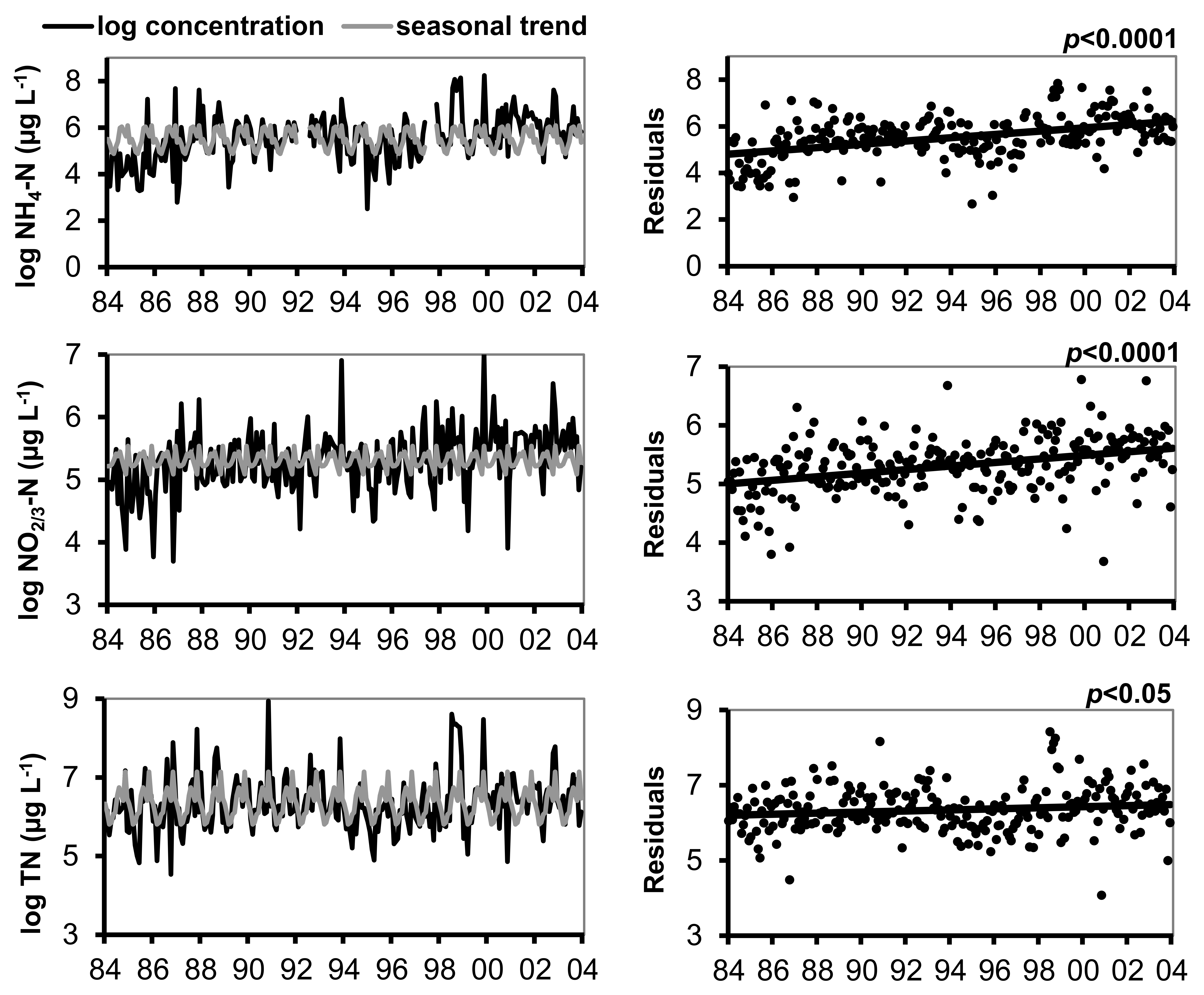

Supplement: Figure S6 — The natural log of nitrogen concentration (µg L−1) in atmospheric deposition at the Flathead Lake Biological Station and the best-fit seasonal trend are shown in the graphs at left. A least squares regression of the deseasonalized data (residuals) against date display the linear trend in the graphs at right. [file peerj-03-841-s013.png]

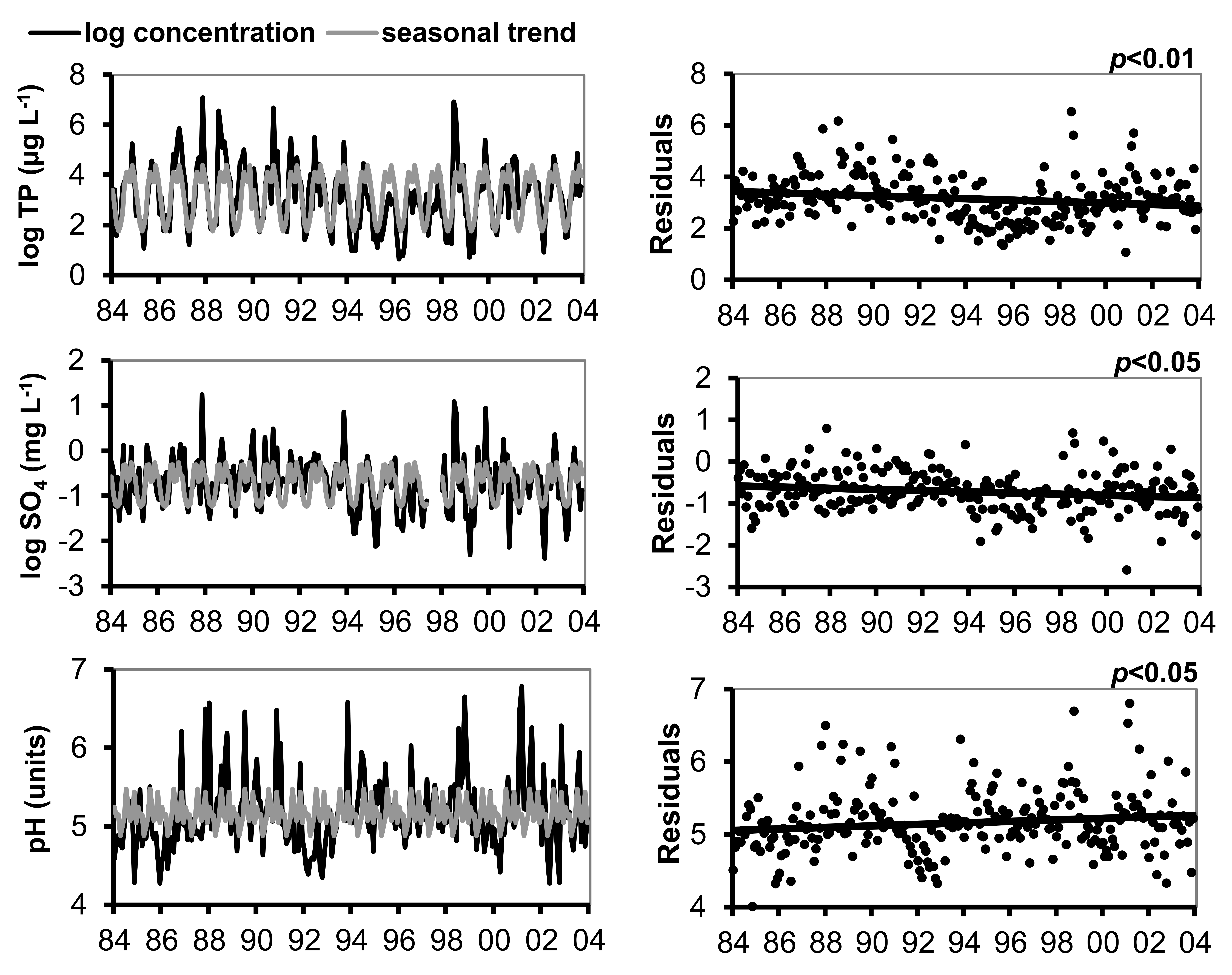

Supplement: Figure S7 — The natural log of TP and SO4 concentrations (µg L−1 and mg L−1, respectively) and pH values in atmospheric deposition at the Flathead Lake Biological Station and the best-fit seasonal trend are shown in the graphs at left. A least squares regression of the deseasonalized data (residuals) against date display the linear trend in the graphs at right. [file peerj-03-841-s014.png]

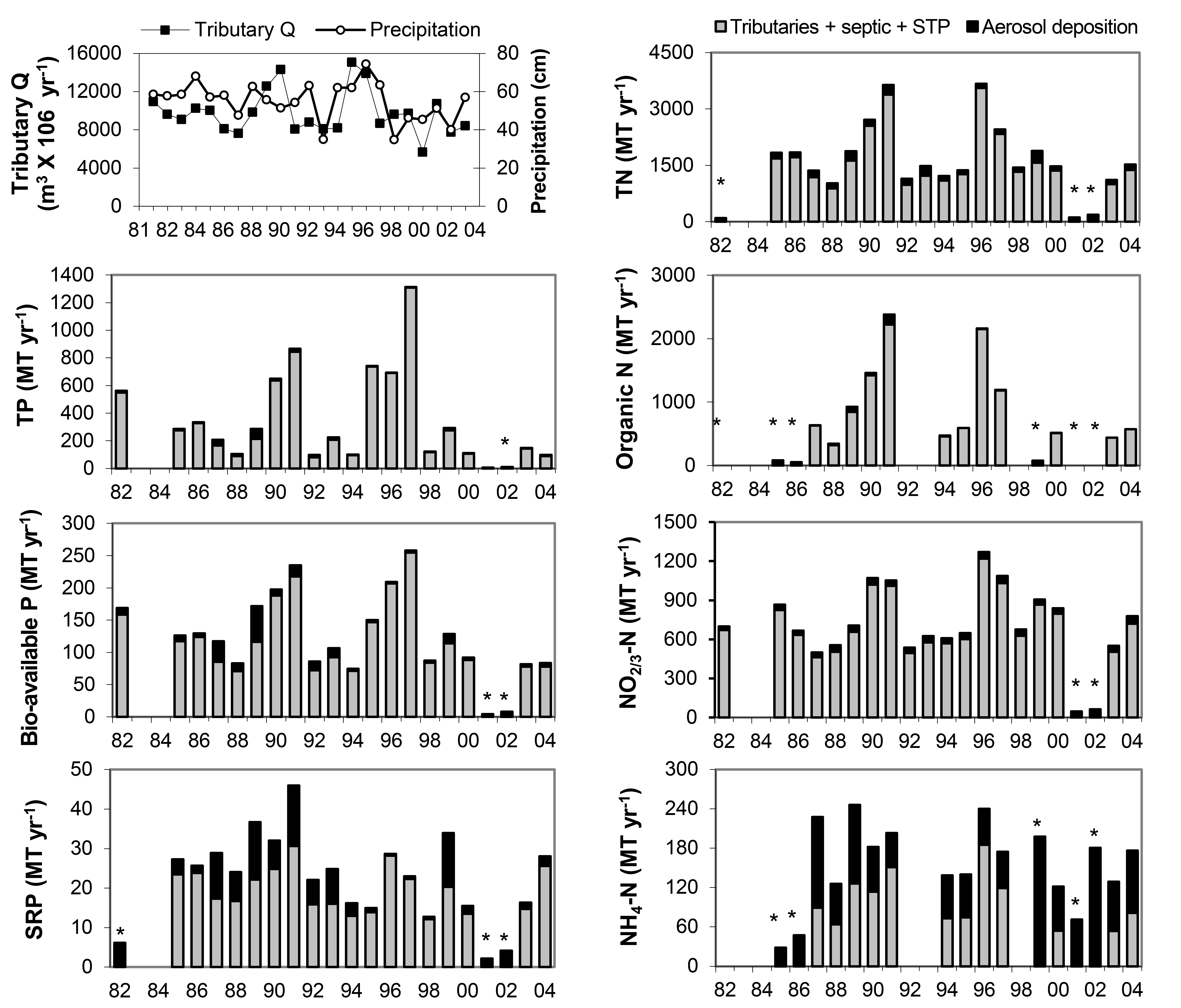

Supplement: Figure S8 — Discharge of the major tributaries to Flathead Lake and precipitation volume at the NOAA site at the Flathead Lake Biological Station by water year are shown in the top left graph. Total nutrient load to the lake from atmospheric deposition (dark portion of histogram) and tributary plus septic system and sewage treatment plant sources (light portion of histogram) are shown in the remaining graphs. Asterisks denote lack of tributary loads for water years when atmospheric loading was determined. Estimates of nutrient loading from septic systems were from Makepeace & Mladenich (1996). Nutrient loading from sewage treatment plants were calculated from daily discharge measurements and monthly nutrient concentrations obtained from Montana Department of Environmental Quality. [file peerj-03-841-s015.png]
